# Single‐Cell Multiomics Reveals TCR Clonotype‐Specific Phenotype and Stemness Heterogeneity of T‐ALL Cells

**DOI:** 10.1111/cpr.13786

**Published:** 2024-12-15

**Authors:** Songnan Sui, Xiaolei Wei, Yue Zhu, Qiuyue Feng, Xianfeng Zha, Lipeng Mao, Boya Huang, Wen Lei, Guobing Chen, Huien Zhan, Huan Chen, Ru Feng, Chengwu Zeng, Yangqiu Li, Oscar Junhong Luo

**Affiliations:** ^1^ Department of Systems Biomedical Sciences, School of Medicine Jinan University Guangzhou China; ^2^ Key Laboratory for Regenerative Medicine of Ministry of Education, Institute of Hematology, School of Medicine Jinan University Guangzhou China; ^3^ Department of Hematology, First Affiliated Hospital Jinan University Guangzhou China; ^4^ Central People's Hospital of Zhanjiang Zhanjiang China; ^5^ Zhanjiang Key Laboratory of Leukemia Pathogenesis and Targeted Therapy Research Zhanjiang China; ^6^ Department of Hematology, Nanfang Hospital Southern Medical University Guangzhou China; ^7^ Department of Clinical Laboratory, First Affiliated Hospital Jinan University Guangzhou China; ^8^ Department of Microbiology and Immunology, Institute of Geriatric Immunology, School of Medicine Jinan University Guangzhou China; ^9^ Guangdong Second Provincial General Hospital, Integrated Chinese and Western Medicine Postdoctoral Research Station, School of Medicine Jinan University Guangzhou China

**Keywords:** clonotype‐specific T‐ALL characteristics, immature thymocyte, single‐cell multiomics, T‐cell acute lymphoblastic leukemia (T‐ALL), T‐cell receptor (TCR) clonotype

## Abstract

T‐cell acute lymphoblastic leukaemia (T‐ALL) is a heterogeneous malignant disease with high relapse and mortality rates. To characterise the multiomics features of T‐ALL, we conducted integrative analyses using single‐cell RNA, TCR and chromatin accessibility sequencing on pre‐ and post‐treatment peripheral blood and bone marrow samples of the same patients. We found that there is transcriptional rewiring of gene regulatory networks in T‐ALL cells. Some transcription factors, such as *TCF3* and *KLF3*, showed differences in activity and expression levels between T‐ALL and normal T cells and were associated with the prognosis of T‐ALL patients. Furthermore, we identified multiple malignant TCR clonotypes among the T‐ALL cells, where the clonotypes consisted of distinct combinations of the same TCR α and β chain per patient. The T‐ALL cells displayed clonotype‐specific immature thymocyte cellular characteristics and response to chemotherapy. Remarkably, T‐ALL cells with an orphan TCRβ chain displayed the strongest stemness and resistance to chemotherapy. Our study provided transcriptome and epigenome characterisation of T‐ALL cells categorised by TCR clonotypes, which may be helpful for the development of novel predictive markers to evaluate treatment effectiveness for T‐ALL.

## Graphical abstract

Delineation of rewired regulatory networks and TCR clonotype‐specific heterogeneity in T‐ALL cells by single‐cell multiomics, providing critical insights for potential therapeutic targets, disease stratification method and prognosis predictor for T‐ALL.

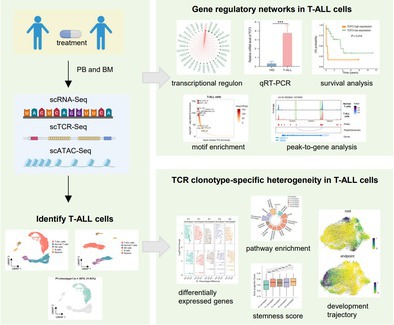

## 1 Introduction

T‐cell acute lymphoblastic leukaemia (T‐ALL) is an aggressive haematological disease characterised by the malignant proliferation of immature T‐cell precursors [1, 2]. It accounts for approximately 25% of adult acute lymphoblastic leukaemia (ALL) [3, 4]. Although intensive chemotherapy leads to high cure rates, the 5‐year survival rate remains under 50%, and patients who experience relapse face a mortality rate of up to 90% [5, 6]. These phenomena are partly caused by the T‐ALL cell heterogeneity, which is fundamentally linked to the complex process of T‐cell differentiation and development [7, 8].

In T‐cell development, several crucial checkpoints exist, including in‐frame TCR β‐chain rearrangement (β‐selection), in‐frame TCR α‐chain rearrangement (α‐selection), positive selection, and negative selection [9, 10, 11]. The initial checkpoint, TCR β‐selection, takes place at the CD4^−^CD8^−^ double negative (DN) stage [12, 13]. Advancement and differentiation beyond the β‐selection checkpoint predominantly depend on the expression of the pre‐TCR [14]. The successful formation of the pre‐TCR complex enables cells to progress through β‐selection [15, 16]. Failure to progress correctly through the β‐selection checkpoint sometimes can lead to T‐ALL [17, 18]. The prevailing belief in T‐ALL is that the earlier T cells undergo malignant transformation during the development process, the stronger their stemness, and the worse their prognosis [19].

During T‐cell development, the majority of T cells progress through multiple rounds of TCR gene recombination [20]. The recombination of the TCRβ chain undergoes allelic exclusion, ensuring that developing T lymphocytes assemble a functional TCRβ gene on a single allele [21]. Conversely, allelic exclusion does not strictly apply during TCRα chain rearrangement [22], which simultaneously occurs on both alleles [23, 24]. Multiple recombination events during T‐cell development can lead to the generation of up to three or four chains within a single cell. Studies have observed that approximately 7% of T cells could display two productive β‐chains, and about 1% express both of these chains on the cell surface [25, 26]. In comparison, about 7% to 30% of T cells express two functionally active α chains [27, 28]. Nonetheless, the molecular landscape of TCR for T‐ALL cells potentially originated from T‐cell precursors at different developmental stages is still unclear.

Typically, T‐ALL arises from monoclonally expanded malignant T cells [29]. However, studies have reported there was more than one malignant TCR clonotype in T‐cell malignancies [30, 31]. Despite these intriguing discoveries in these leukaemia/lymphoma contexts, there is currently a lack of research focused on the heterogeneity of malignant TCR clonotypes at the single‐cell level in T‐ALL. In this study, we employed scRNA‐seq, scTCR‐Seq and scATAC‐Seq to identify and investigate the heterogeneity of T‐ALL. We explored gene regulatory networks in T‐ALL cells and further investigated the phenotypic, functional, and therapeutic efficacy differences among T‐ALL cells with different TCR clonotypes.

## 2 Results

### 2.1 Identification of T‐ALL Cells With Distinct TCR Clonotype

**FIGURE 1:**
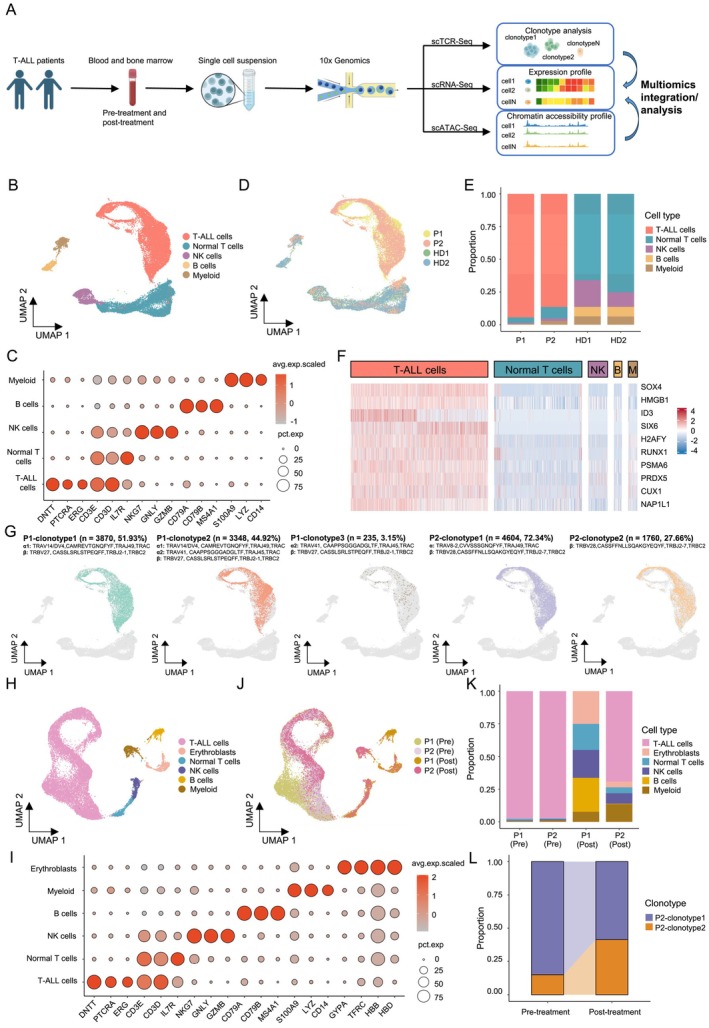
Identification of T‐ALL cells in PBMCs and BMMCs from T‐ALL patients by scRNA‐Seq and scTCR‐Seq. (A) Study design schematics. (B) UMAP (Uniform Manifold Approximation and Projection) plot of PBMC scRNA‐seq datasets from two T‐ALL patients and two healthy donors, colour‐coded by five distinct cell types. (C) Dot plot of marker genes for each cell type in PBMCs. Colour scale shows the average normalised expression of marker genes in each cell type, and dot size indicates the percentage of cells within each cell cluster expressing the marker gene. The same applies to all other dot plots in this paper. (D) Same as (B), but colour‐coded by sample origin. P1: Patient1; P2: Patient2; HD1: Healthy donor 1; HD2: Healthy donor 2. (E) Stacked bar chart for summarising the proportion of cell types in PBMCs of each T‐ALL patient and normal donor, according to the scRNA‐Seq datasets. (F) Heatmap of expression levels of selected transcription factors associated with blood malignancy. (G) Same as (B), but colour‐coded by individual TCR clonotype. Patient origin, clonotype ID, clonotype frequency and actual TCRαβ clonotype are shown on top of each UMAP. (H) UMAP plot of BMMC scRNA‐seq dataset from two T‐ALL patients pre‐ and post‐treatment, colour‐coded by six distinct cell types. (I) Dot plot of marker genes for each cell type in BMMCs. (J) Same as (H), but colour‐coded by patient origin and treatment condition. Pre: Pre‐treatment; Post: Post‐treatment. (K) Stacked bar chart for summarising the proportion of cell types in BMMCs of each T‐ALL patient and treatment condition. (L) Comparison of the proportions of T‐ALL cells with two distinct TCR clonotype from Patient2 (P2), pre‐ and post‐treatment.

To characterise the transcriptome profile and TCR clonotypes of T‐ALL tumour cells at single‐cell resolution, we collected blood mononuclear cells (PBMCs) and bone marrow mononuclear cells (BMMCs) from a newly diagnosed T‐ALL patient (Patient1, P1) and a relapsed T‐ALL patient (Patient2, P2), pre‐ and post‐treatment for scRNA‐Seq and scTCR‐Seq analysis (Figure 1A). After chemotherapy, P1 resulted in a complete response (CR), while P2 ended in non‐remission (NR).

Following data quality control, we integrated the pre‐treatment PBMC scRNA‐Seq datasets obtained from the two T‐ALL patients with previously published PBMC scRNA‐Seq datasets from two healthy donors (Table S1) for cell type delineation. We identified five major cell clusters annotated as T‐ALL, normal T, NK (natural killer), B, and myeloid cells, according to the corresponding marker genes (Figure 1B,C; Figure S1). Notably, the non‐malignant cell clusters were identified in both T‐ALL patients and healthy donors, whereas T‐ALL cells were exclusive to patients (Figure 1D). Moreover, the patient‐derived PBMCs predominantly consisted of T‐ALL cells, with non‐malignant cells taking up significantly lower proportions in the total cell population (Figure 1E). To further verify the identified T‐ALL cell cluster, we employed a random‐forest model to screen for TF genes that could effectively distinguish malignant cells from non‐malignant cells. The results revealed several TF genes, such as *SOX4*, *RUNX1* and *MYB*, known to be related to T‐ALL, were among the top contributing genes for separating T‐ALL cells from the rest (Figure S1). Furthermore, the top‐ranked TF genes from the random‐forest model had higher expression in T‐ALL patients compared to healthy donors in both our and a previously published dataset (Figure 1F; Figure S1).

Next, we examined the TCR clonotype of the T‐ALL cells. The TCRs of T‐ALL cells were clonally expanded with much higher frequency compared to the normal T cells in these patients (Table S2). Only five TCR clonotypes were detected on the T‐ALL cells from these two assayed patients, with TCRs from the T‐ALL cells of the same patient sharing a unique patient‐specific TCR chain (Figure 1G). Specifically, T‐ALL cells of P1 were of three TCR clonotypes, of which P1‐clonotype1 (TCRα1β, 51.93%) and P1‐clonotype3 (TCRα2β, 3.15%) had the identical TCRβ chain but different TCRα, and the third clonotype (P1‐clonotype2: TCRα1α2β, 44.92%) also had the same TCRβ, however, paired with the two TCRα chains from P1‐clonotype1 and P1‐clonotype3 (Figure 1G). For the second patient, the T‐ALL cells were of two TCR clonotypes, P2‐clonotype1 (72.34%) and P2‐clonotype2 (27.66%) with identical TCRβ. Nevertheless, the minority P2‐clonotype2 T‐ALL cells in this patient only had a TCRβ chain, without any identifiable TCRα, at the RNA level (Figure 1G). scRNA‐Seq and scTCR‐Seq of thymocytes at different stages have also revealed the presence of thymocytes expressing a single TCRβ chain and T cells expressing two TCRα chains [32, 33]. These findings indicate that T‐ALL is not characterised by monoclonal expansion. Instead, there may be different TCR clonotypes in T‐ALL cells, with diverse TCRα and TCRβ combinations unique to specific patients.

Similar to the pre‐treatment results, the post‐treatment PBMCs from the two patients also consisted of the same five major cell types: T‐ALL, normal T, NK, B and myeloid cells (Figure S1). However, the prevalence of T‐ALL cells dramatically reduced, and was only present in P2, who had not achieved remission (Figure S1).

Next, we conducted integrated analysis on the pre‐ and post‐treatment BMMC scRNA‐Seq datasets to compare changes in T‐ALL cells before and after treatment. We were able to identify six distinct cell clusters in bone marrow (BM) based on the expression of marker genes (Figure 1H,I). Before treatment, the BM was predominantly occupied by T‐ALL cells (Figure 1J,K), consistent with our earlier findings in the peripheral blood (PB). The same previously identified TCR clonotypes of T‐ALL cells were re‐identified in the BMMC samples, with remarkably similar proportions among T‐ALL cells in BM compared to PB samples (Figure S1). In the post‐treatment BMMCs, we observed a notable increase in non‐malignant cells (Figure 1J,K). In P1, the transcriptome and TCR profiling collectively indicated that all tumour cells were eradicated, resulting in the absence of T‐ALL cells post‐treatment (Figure 1K; Figure S1). Conversely, in P2, different from the PBMC sample, there was still a high amount of T‐ALL cells in the post‐treatment BM (Figure 1J,K). In particular, a higher proportion of T‐ALL cells with P2‐clonotype2 TCR persisted in the post‐treatment BM compared to pre‐treatment (Figure 1L; Figure S1). These findings suggest that T‐ALL cells with distinct clonotypes may be phenotypically different and respond differently to therapy.

### 2.2 Single‐Cell Chromatin Accessibility Profiling of T‐ALL

**FIGURE 2:**
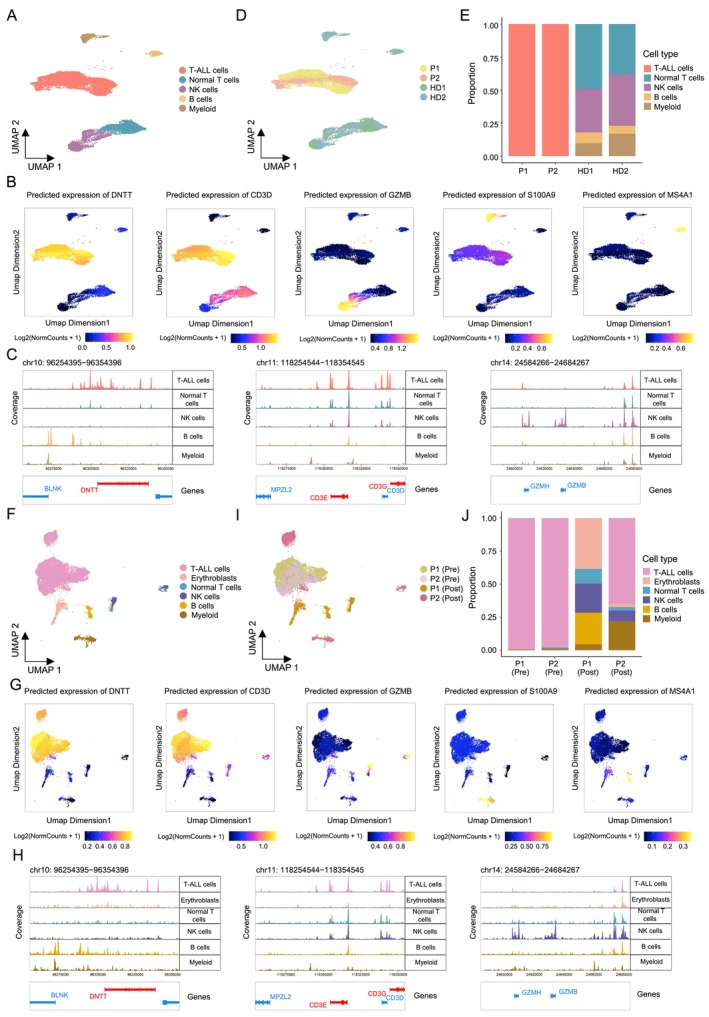
Characterisation of T‐ALL cells in PBMC and BMMC of T‐ALL patients by scATAC‐Seq. (A) UMAP plot of PBMC scATAC‐seq datasets from two T‐ALL patients and two healthy donors, colour‐coded by five identified cell types. (B) Projection of gene scores of selected marker genes on PBMC scATAC‐Seq UMAP visualisation. Gene score reflects predicted expression of the corresponding gene, higher score implies higher accessibility. (C) Track view of the PBMC scATAC‐Seq data for selected loci in distinct cell types. (D) Same as (A), but colour‐coded by sample origin. (E) Stacked bar chart for summarising the proportion of cell types in PBMCs of each T‐ALL patient and normal donor, according to the scATAC‐Seq datasets. (F) UMAP plot of BMMC scATAC‐seq datasets from two T‐ALL patients pre‐ and post‐treatment, colour‐coded by six identified cell types. (G) Projection of gene scores of selected marker genes on BMMC scATAC‐Seq UMAP visualisation. (H) Track view of the BMMC scATAC‐Seq data for selected loci in distinct cell types. (I) Same as (F), but colour‐coded by sample origin with different treatment condition. P1: Patient1; P2: Patient2; Pre: Pre‐treatment; Post: Post‐treatment. (J) Stacked bar chart for summarising the proportion of cell types in BMMCs of each T‐ALL patient pre‐ and post‐treatment, according to the scATAC‐Seq datasets.

In addition to scRNA‐Seq, we also conducted scATAC‐Seq to gain insights into the chromatin accessibility of T‐ALL cells. After data quality control and filtering (Table S3), combinatorial analysis of scATAC‐Seq datasets of pre‐treatment PBMC samples from the two patients and two healthy donors identified five cell clusters (Figure 2A). By utilising the previously described PBMC scRNA‐Seq datasets, and the predicted gene expression profiles based on promoter accessibility, we were able to accurately infer and annotate the cell types of these five cell clusters in scATAC‐Seq data (Figure 2A–C; Figure S2). Consistent with the scRNA‐Seq results, PBMC scATAC‐Seq datasets from T‐ALL patients pre‐treatment were predominantly from T‐ALL cells (Figure 2D,E). Likewise, we successfully identified six distinct cell clusters in BMMC samples from the patient's pre‐ and post‐treatment (Figure 2F–H; Figure S2). Similar to the scRNA‐Seq data, pre‐treatment BMMCs were almost entirely composed of T‐ALL cells, while post‐treatment BMMCs exhibited an increased proportion of non‐malignant cells (Figure 2I,J). Furthermore, the BMMC scATAC‐Seq data from the patients revealed consistent patient‐specific pre‐ and post‐treatment cell type composition dynamics with the scRNA‐Seq data.

### 2.3 Transcriptional Regulation Rewiring in T‐ALL Cells

**FIGURE 3:**
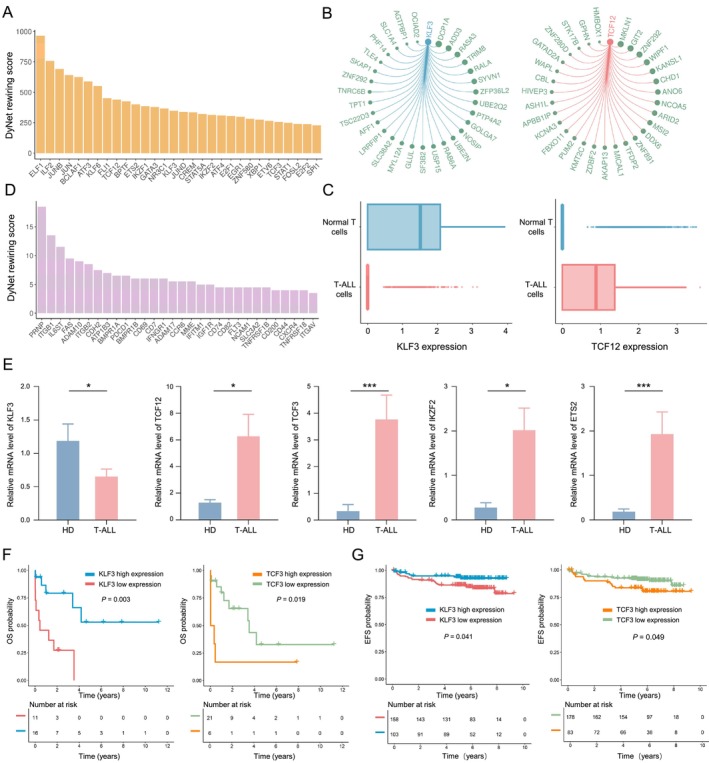
T‐ALL cells specific gene regulatory programs. (A) TFs arranged based on DyNet scores calculated from separate gene regulatory networks in T‐ALL cells and normal T cells. Higher DyNet score implies higher rewiring score for the same regulator (TF or surface protein) in different cell types. (B) Circos plots showing exemplary regulons in normal T cell and T‐ALL cell, respectively. Normal T‐cell‐specific and T‐ALL cell‐specific TF is shown in blue and red, respectively, with links connecting to the target genes. Top 30 target genes are shown for each TF. Node size of target genes is proportional to regulation weight by the corresponding TF. (C) Boxplots comparing the expression levels of *KLF3* (left) and *TCF12* (right) between normal T cells and T‐ALL cells. For the boxplots, the outlines of the boxes represent the first and third quartiles, the line inside each box represents the median, and boundaries of the whiskers are found within 1.5 times the interquartile range, with dots representing outliers. The same applies to all other boxplots in this manuscript, except stated otherwise. (D) Cell surface proteins arranged based on DyNet scores calculated from separate gene regulatory networks in T‐ALL cells and normal T cells. (E) Comparison of KLF3, TCF12, TCF3, IKZF2 and ETS2 expression levels in healthy donor (HD) and T‐ALL patients. *p < 0.05, **p < 0.01, ***p < 0.001 (Wilcoxon rank‐sum test, two‐sided). (F) OS analysis of KLF3 and TCF3 in T‐ALL patients from JNU dataset. (G) EFS analysis of KLF3 and TCF3 in T‐ALL patients from TARGET database.

Gene regulatory networks play a crucial role in haematopoiesis and related malignancy [34, 35, 36]. We sought to investigate whether genes are regulated differently in T‐ALL cells compared to normal T cells. First, using the pre‐treatment PBMC scRNA‐Seq data, we identified 303 and 265 activated regulons in T‐ALL and normal T cells, respectively. Among them, 153 regulons were commonly activated in both cell types. Then, we compared the active regulons between T‐ALL and normal T cells to understand how regulatory relationships between TFs and target genes were rewired due to malignancy [37] (Table S4). We firstly focused on the TFs with the most significant alterations in their connections to target genes (Figure 3A). Many of these TFs with highly rewired regulatory activity were closely associated with T‐cell development. For instance, *KLF3* and *KLF6* exhibited specific transcription activation functions in normal T cells, whereas strong activation functions by *TCF12* and *TCF3* were uniquely observed in T‐ALL cells (Figure 3B; Figure S3). Interestingly, some TFs, such as *ETS2* and *IKZF2*, were of strong regulatory activity in both cell types, but some target genes were uniquely gained or lost in T‐ALL cells compared to normal T cells (Figure S3). Notably, *KLF3* and *KLF6* exhibited significantly higher expression in normal T cells, whereas *TCF12*, *TCF3*, *ETS2*, and *IKZF2* displayed higher expression in T‐ALL cells (Figure 3C; Figures S3). Moreover, we identified the cell surface marker genes with the most prominently rewired activation in T‐ALL, which could be used as potential therapeutic targets (Figure 3D). For instance, among the top actively rewired cell surface marker genes, *CD7* has already been utilised as a target in chimeric antigen receptor T‐cell (CAR‐T) therapy for refractory/relapsed T‐ALL, and *CXCR4* monoclonal antibodies were also used in a clinical trial for refractory/relapsed T‐ALL [38, 39, 40].

Next, we used RNA from PBMCs of T‐ALL patients and normal T cells from healthy donors to validate the gene expression levels of the TFs via quantitative reverse transcription polymerase chain reaction (qRT‐PCR). We found that *KLF3* was significantly higher in healthy donors compared to T‐ALL patients, while the expression levels of *TCF12*, *TCF3*, *ETS2*, and *IKZF2* were markedly elevated in T‐ALL patients (Figure 3E). Furthermore, survival analysis revealed that T‐ALL patients with higher *KLF3* expression levels had longer overall survival (OS) and better prognosis, whereas T‐ALL patients with lower *TCF3* expression levels showed shorter OS and poorer prognosis (Figure 3F). Additionally, survival analysis of paediatric T‐ALL patients from the TARGET database showed that those with high *KLF3* expression had longer event‐free survival (EFS), while those with low *TCF3* expression also had longer EFS (Figure 3G). Further analysis of the co‐expression status of *KLF3* and *TCF3* impacting the prognosis of T‐ALL patients revealed that T‐ALL patients with high expression of *KLF3* and low expression of *TCF3* had the best prognosis (Figures S3).

**FIGURE 4:**
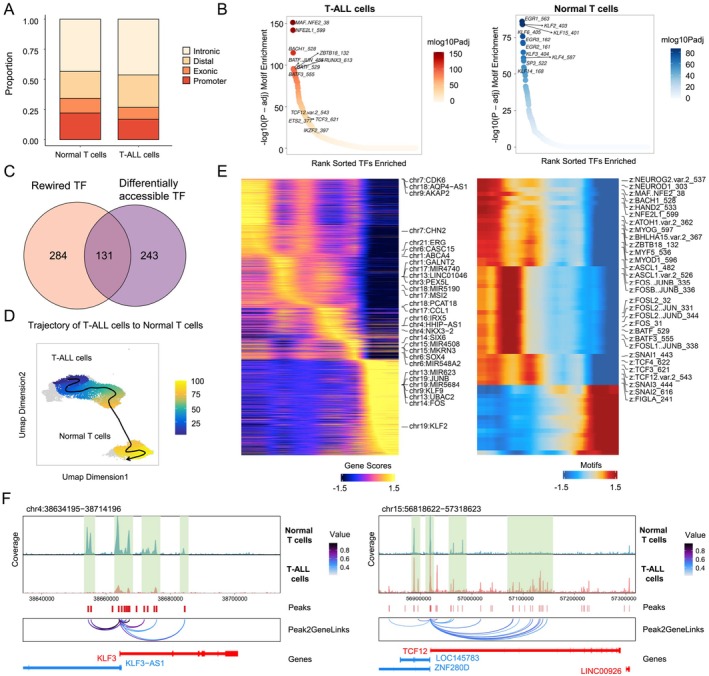
Gene regulatory programs associated changes in chromatin accessibility in T‐ALL cells. (A) Stacked barplot of different types of peaks in normal T cells and T‐ALL cells. (B) Ranking of TF binding motifs enriched in chromatin accessibility peaks identified in T‐ALL cells (left) and normal T cells (right). (C) Venn diagram indicating the overlap between differentially enriched TF binding motifs and rewired TFs identified by the DyNet algorithm between T‐ALL cells and normal T cells. (D) Pseudotime trajectory spanning from normal T cells to T‐ALL cells based on chromatin accessibility. (E) Heatmap displaying the changes of gene scores (left) and TF motif accessibility (right) along the pseudotime trajectory from normal T cells to T‐ALL cells. (F) Track view of scATAC‐Seq data, chromatin accessibility peaks and predicted peak‐to‐gene interaction of the *KLF3* (left) and *TCF12* locus (right).

We then sought to compare the chromatin accessibility landscape between T‐ALL and normal T cells, in an effort to understand the epigenetic mechanism of differential gene regulatory activities. We identified 59,552 and 90,187 significant chromatin accessibility peaks in normal T and T‐ALL cells, respectively (Figure S4). Peak annotation revealed a higher proportion of distal open chromatin regions in T‐ALL cells compared to normal T cells (Figure 4A), and T‐ALL cells were of a much greater number of differentially enhanced open chromatin regions (Figure S4). The open chromatin regions in T‐ALL were located further away from the nearest gene transcription start site (TSS) (Figure S4), and the promoters in T‐ALL were predicted to be linked with a higher number of distal open chromatin regions (i.e., enhancers) (Figure S4). Then, we identified the enrichment of distinct TF binding motifs in the normal T and T‐ALL cell‐specific open chromatin regions, respectively. For example, binding motifs of *KLF3* and *KLF6* were significantly enriched in the normal T‐cell‐specific open chromatin regions, whereas *TCF13*, *TCF3*, *ETS2*, and *IKZF2* motifs were highly enriched for T‐ALL cell (Figure 4B).

Among the total of 415 TFs with rewired regulatory activity between normal and T‐ALL cells, we found 131 of them were also with binding motifs enriched in the cell‐type‐specific open chromatin regions (Figure 4C). This potentially implies that the differential open chromatin regions with distinct TF motif enrichment contributed to the rewiring of gene regulatory networks. Next, we performed chromatin accessibility pseudotime trajectory analysis using the scATAC‐Seq data from normal T and T‐ALL cells, and the results revealed a clear transition trajectory from T‐ALL cells to normal T cells (Figure 4D). Moreover, genes known to be associated with T‐ALL, including *ERG* and *CDK6*, had high to low predicted gene expression along the trajectory (Figure 4E). Additionally, TF binding motif enrichment along the trajectory once again indicated binding motifs of *TCF3* and *TCF12* were preferentially enriched in open chromatin regions of T‐ALL cells (Figure 4E). Lastly, we compared the accessibility of predicted distal regulatory elements linked to the cell‐type‐specific TF gene promoter. It is evident that the promoters of *KLF3* and *KLF6* were linked to more active enhancers in normal T cells, whereas promoters of T‐ALL specific TF genes (e.g., *TCF12*, *TCF3*, *ETS2*, and *IKZF2*) were associated with more active enhancers in T‐ALL cells (Figure 4F; Figure S4). These results collectively revealed T‐ALL cell‐specific chromatin accessibility and gene regulatory characteristics potentially associated with T‐ALL malignancy.

### 2.4 Distinctive TCR Clonotype‐Specific Characteristics of T‐ALL Cells

**FIGURE 5:**
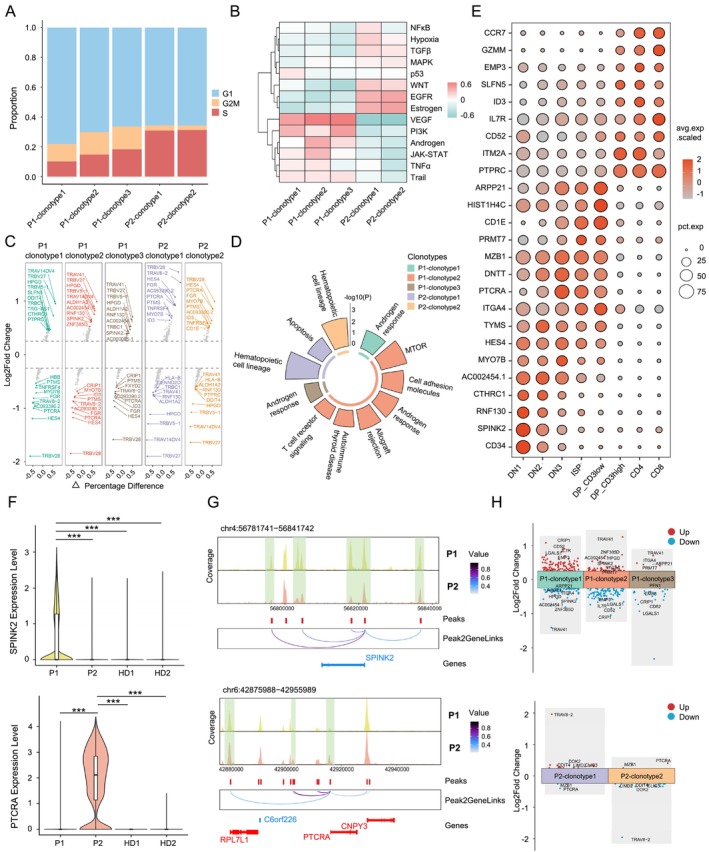
TCR clonotype‐specific characteristics of T‐ALL cells. (A) Proportion of cell cycle phases in each TCR clonotype depicted by a stacked bar chart. (B) Heatmap illustrating heterogeneity of tumour‐associated signalling pathway activity. (C) Volcano plot for marker genes of the five different TCR clonotypes. (D) Enriched pathways for the upregulated genes in the T‐ALL cells with five different TCR clonotypes. (E) Dot plot depicting the mean expression levels and cell expression proportions of marker genes for thymocytes from different differentiation stages. (F) Comparison of the *SPINK2* (top) or *PTCRA* (bottom) expression levels in each sample. ***p < 0.001 (Wilcoxon rank‐sum test, two‐sided). (G) Browser tracks showing the chromatin accessibility profile and peak‐to‐gene links at the *SPINK2* locus (top) or *PTCRA* locus (bottom) across T‐ALL cells of P1 and P2. (H) Scatterplot showing the log2(Fold Change) of marker genes expression of the three different TCR clonotypes in P1 (top) or two different TCR clonotypes in P2 (bottom).

Although clustering analysis on the scRNA‐Seq and scATAC‐Seq data grouped all T‐ALL cells into one cluster, suggesting similar transcriptional profiles and chromatin accessibility landscape among all T‐ALL cells, it is essential to consider the potential differences among T‐ALL cells with different TCR clonotypes. Firstly, we predicted the cell cycle stage in which the T‐ALL cells were based on the scRNA‐Seq data. Specifically, the T‐ALL cells from P2 with two distinct TCR clonotypes were of a similar but higher proportion of actively dividing (S phase) cells comparing to T‐ALL cells from P1; the T‐ALL cells from P1 with TCR clonotype of P1‐clonotype1, P1‐clonotype2 and P1‐clonotype3 had increasing proportion of S phase cells (Figure 5A). Additionally, we assessed the activity of tumour‐associated signalling pathways and observed significant heterogeneity among T‐ALL cells with different TCR clonotypes (Figure 5B).

The T‐ALL cells with distinct TCR clonotypes were of specific differentially expressed genes (Figure 5C), and functional enrichment analysis of the upregulated genes in each clonotype‐specific T‐ALL cell revealed interesting patterns (Figure 5D; Table S5). All T‐ALL cells with the three distinct clonotypes in P1 were enriched for genes in the androgen response pathway, while only the P1‐clonotype2 (TCRα1α2β) T‐ALL cell group especially showed functional enrichment in the TCR signalling pathway. The T‐ALL cell groups in P2 with the two clonotypes both had upregulated expression of gene enriched in the haematopoietic cell lineage pathway, and the P2‐clonotype1 group additionally showed enriched gene expression in the apoptosis pathway (Figure 5D; Table S5). Some of the clonotype‐specific T‐ALL expressed genes including *SPINK2*, *AC002454.1*, *PTCRA*, and *HES4*, which were also recognised as marker genes for thymocytes at different differentiation stages (Figure 5C,E). For example, T‐ALL cells in P1 presented a high expression level of *SPINK2*, while only P2 had a high expression level of *PTCRA* (Figure 5F). These findings were further supported by the scATAC‐Seq data, which revealed higher promotor accessibility for *SPINK2* and *PTCRA* in T‐ALL cells of P1 and P2, respectively (Figure 5G). We also identified intra‐patient differentially expressed genes among T‐ALL cells with distinct clonotypes. The upregulated genes in P1‐clonotype2 and P1‐clonotype3 groups, such as *SPINK2*, *AC002454.1*, and *ARPP21*, were also expressed during early stages (DN, ISP and DP_CD3low) of thymocyte differentiation (Figure 5E,H; Figure S5). In contrast, upregulated genes in P1‐clonotype1 cells including *EMP3* and *CD52* were highly expressed during late stages (DP_CD3high and SP) of thymocyte development (Figure 5E,H). As for P2, *MZB1* and *PTCRA* with the upregulated expression in the P2‐clonotype2 group were also highly expressed during early differentiation stages (DN, ISP and DP_CD3low) of thymocytes (Figure 5E,H; Figure S5). These results suggest the T‐ALLs with distinct TCR clonotypes were situated at distinct thymocyte differentiation stages.

Furthermore, we sought to explore potential differences between T‐ALL cells in PB and the ones in BM. Upon integrating the scRNA‐Seq datasets from PBMCs and BMMCs per patient, we observed only minimal differences (Figure S5). In P1, T‐ALL cells from BM, compared to PB, exhibited only one upregulated and downregulated gene, respectively (Figure S5). The same phenomenon was maintained across different groups of T‐ALL cells with distinct TCR clonotypes in P1 (Figure S5). Moreover, similar chromatin accessibility profiles were observed between T‐ALL cells derived from BM and PB, indicated by the similar proportion of chromatin accessibility peak annotations and very few differential peaks (Figure S5).

### 2.5 Cell Stemness Heterogeneity Among T‐ALL Cells With Different TCR Clonotypes

**FIGURE 6:**
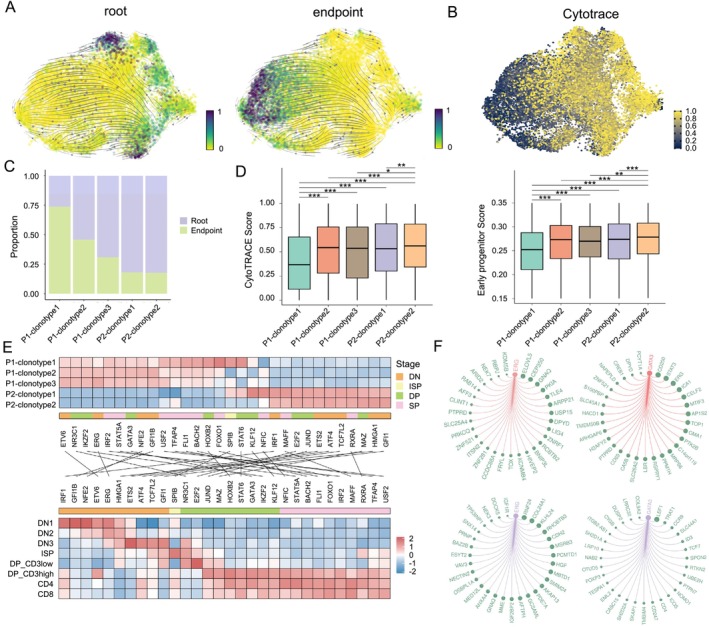
Comparison of stemness among T‐ALL cells of five distinct TCR clonotypes. (A) UMAP plot highlighting the RNA velocity results, specifically emphasising root and endpoint cells. (B) Distribution of differentiation states, ranging from the highest values representing the most immature state to the lowest values representing the most mature state, as determined by CytoTRACE. (C) Proportion of root cells and endpoint cells in each clonotype. (D) Box plots for comparing the T‐ALL cell CytoTRACE score (left) and early progenitor score (right) among the five different TCR clonotypes. *p < 0.05; **p < 0.01; ***p < 0.001 (Wilcoxon rank‐sum test, two‐sided). (E) Heatmap displaying the normalised activity of 30 marker transcription factor (TF) regulons in the five different TCR clonotypes as determined by pySCENIC (top). Heatmap displaying the normalised activity of the identified TF regulons in the top heatmap based on scRNA‐Seq dataset from thymocytes originating from different differentiation stages. (F) Regulons containing TFs and their top 30 targets activated in T‐ALL cells (top) and thymocytes (bottom) such as *ERG* and *GATA3*.

The entire process of TCR rearrangement occurs at different stages of T‐cell development and differentiation [10]. As described above, the assayed T‐ALL cells with different TCR clonotypes were of specific genes also expressed in thymocytes at different differentiation stages. Therefore, the T‐ALL cells with different TCR clonotypes may present varying degrees of stemness. To investigate this, we carried out RNA velocity analysis on T‐ALL cells, which defined root and endpoint cells (Figure 6A). The root cells were enriched for the haematopoietic stem cell pathway, while endpoint cells were enriched for differentiating the T‐lymphocyte and interferon alpha pathway (Figure S6). Additionally, cell cycle mapping revealed a higher proportion of dividing cells (S phase) in the root cells compared to the endpoint cells (Figure S6). Upregulated genes in the root cells were closely related to early stages of thymocyte differentiation, such as *DNTT*, *HES4*, and *PTCRA*. On the other hand, endpoint cells were marked by high expression of *CD52* and *CCR7*, which were associated with late stages of thymocyte differentiation (Figure S6). Furthermore, according to CytoTRACE [41] analysis for cell stemness prediction, the root cells were found to possess a higher developmental potential compared to the endpoint cells (Figure 6B).

Among the T‐ALL cells with distinct TCR, P2‐clonotype1 and P2‐clonotype2 T‐ALL cells showed an equally higher proportion of cells classified as root cells than the ones from P1 (Figure 6C). The T‐ALL cells from P1 with the three different TCR clonotypes were composed of varying proportions of root and endpoint cells, respectively, with cells with P1‐clonotype1 predominantly annotated as endpoint cells (Figure 6C). Moreover, CytoTRACE scoring indicated P2‐clonotype2 cells exhibited the highest degree of stemness (i.e., high CytoTRACE score), while P1‐clonotype1 cells had the lowest cell stemness (i.e., low CytoTRACE score) (Figure 6D). Similar results were obtained with the early progenitor cell function scoring (Figure 6D). Interestingly, regulatory network analysis revealed that most of the TFs and regulons activated in P2‐clonotype1 and P2‐clonotype2 cells were associated with early‐stage thymocyte development, while TFs and regulons activity in P1 tended to be associated with late‐stage thymocytes (Figure 6E). Specifically, for P1, P1‐clonotype1 cells showed the highest activity of regulons mapped to late‐stage thymocytes. Transcription regulation rewiring was also found between T‐ALL cells and normal thymocytes (Figure 6F). Thus, we deduced that the T‐ALL cells from P2 were of stronger cell stemness compared to the ones from P1, and in particular, T‐ALL cells with P2‐clonotype2 TCR clonotype, which only has an orphan TCRβ chain, were of the highest cell stemness.

### 2.6 Treatment‐Related Changes in Stemness of T‐ALL Cells

**FIGURE 7:**
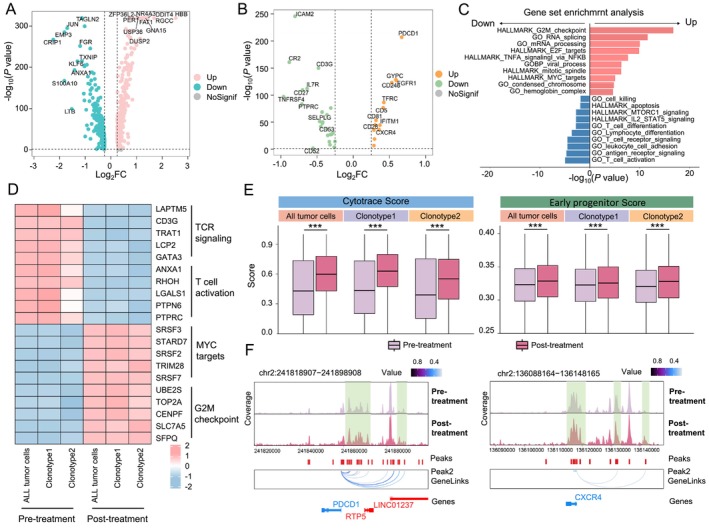
Pre‐ and post‐treatment T‐ALL cell comparison. (A) Volcano plots showing differentially expressed genes between pre‐ and post‐treatment T‐ALL cells. (B) Volcano plots showing differentially expressed surface protein genes between pre‐ and post‐treatment T‐ALL cells. (C) Functional enrichment of up‐ or down‐regulated genes in post‐treatment T‐ALL cells. (D) Heatmap displaying the expression fold‐changes for genes associated with TCR signalling, T‐cell activation, MYC targets, and G2M checkpoint pathways in T‐ALL cells pre‐ and post‐treatment. (E) Boxplots comparing CytoTRACE score (left) and early progenitor score (right) between T‐ALL cells pre‐ and post‐treatment. (F) Browser tracks showing the chromatin accessibility profile and peak‐to‐gene links at the *PDCD1* locus (left) and *CXCR4* locus (right) across T‐ALL cells pre‐ and post‐treatment of Patient2.

After chemotherapy, T‐ALL cells in P2 showed upregulated expression of genes such as *RGCC* and *NR4A3* (Figure 7A; Figure S7). Additionally, cell surface markers including *PDCD1*, *CD5*, and *CXCR4* were also upregulated (Figure 7B; Figure S7). Functional enrichment analysis of these upregulated genes revealed enrichment in MYC_targets and G2M_checkpoint pathways. Meanwhile, genes annotated to pathways associated with T‐cell activation, T‐cell differentiation, and TCR signalling were downregulated in T‐ALL cells post‐treatment (Figure 7C,D). A previous study has proven that *PDCD1* is a marker gene for T‐ALL stem cells, and PD1^+^ T‐ALL cells showed high activity of MYC signalling and low activity of the TCR signalling pathway. For P2 with NR, the CytoTRACE scores and early progenitor cell signature scores of T‐ALL cells in the post‐treatment sample also increased, further supporting the notion of stronger stemness phenotype of T‐ALL cells post‐treatment (Figure 7E). Lastly, the promoters of *PDCD1*, *CXCR4*, *RGCC*, and *NR4A3* all showed higher accessibility in T‐ALL cells post‐treatment (Figure 7F; Figure S7). These results potentially imply that, in the case of NR, though drug treatment eliminates some T‐ALL cells, malignant cells with high cell stemness persisted, thus resulting in higher overall post‐treatment T‐ALL cell stemness.

## 3 Discussion

There is currently a lack of clear understanding about the origin of T‐ALL cells with respect to the thymocyte developmental stage at which malignancy occurs. In this study, we performed multiomics analysis including scRNA‐seq, scTCR‐Seq, scATAC‐seq, and whole genome sequencing, to investigate the transcriptional and chromatin accessibility landscapes of T‐ALL cells pre‐ and post‐treatment. Remarkably, our analyses unveiled the presence of T‐ALL cells with multiple TCR clonotypes in patients at the single‐cell level. Although the T‐ALL cells exhibited similarities in their transcriptional profiles, there are notable differences in terms of phenotype, function, and drug response among cells with different TCR clonotypes, resulting in different prognoses.

We identified the presence of multiple TCR clonotypes in T‐ALL, supporting previous reports of the existence of more than one malignant clonotype in T‐ALL [42]. Utilising single‐cell sequencing, we were able to determine the exact TCR clonotypes of T‐ALL cells, which consisted of multiple modes of combination of the same TCR α and β chain per patient. Of interest, we observed T‐ALL cells with a single chain and TCRs sharing the same β chain but paired with distinct or even double α chains. These malignant TCRs resembled the TCR expression and assembly process through thymocyte development [43], hinting the T‐ALL cells originated from T‐cell precursors situated at distinct development stages. Moreover, we found similarities in the transcriptional profiles among different T‐ALL cells with distinct TCR clonotypes, suggesting potential interconnections between them. It is plausible that during the malignant proliferation of T‐ALL cells, ongoing rearrangements of TCR genes lead to the emergence of new clonotypes, thereby increasing the diversity and heterogeneity of T‐ALL cells.

Analyses of T‐ALL cells with distinct TCR clonotypes within and between patients revealed significant differences in various aspects, including cell cycle regulation, expression modules, tumour‐related signalling pathways, and marker genes. TCRβ rearrangement occurs in the DN stage, while TCRα rearrangement takes place in the DP stage, with both alleles of the α chain undergoing multiple rearrangements simultaneously. Therefore, the T‐ALL cells in P2 with a single‐β chain TCR clonotype (P2‐clonotype2) and a paired αβ chain TCR clonotype (P2‐clonotype1) may become malignant earlier than the T‐ALL cells in P1, which included two kinds of paired αβ chains TCR clonotypes (P1‐clonotype1, P1‐clonotype3) and a triple‐chain TCR clonotype (P1‐clonotype2: TCRα1α2β). Generally, the earlier the malignancy occurs in cells, the stronger the stemness, drug resistance, and poorer prognosis [44, 45]. In this study, we have provided evidence that T‐ALL cell stemness varies significantly among the T‐ALL cells with different TCR clonotypes, and this heterogeneity is closely associated with therapeutic response and prognosis. We observed that T‐ALL cells with TCR featuring a single‐β chain displayed the strongest stemness and, unfortunately, the least favourable chemotherapy outcomes. This discovery confirms the three‐way connection between TCR clonotype, T‐ALL cell stemness and treatment outcome, and opens possibilities for developing a new predictive indicator to assess therapeutic efficacy and prognosis in T‐ALL.

Comparing the clonal evolution of T‐ALL cells between pre‐ and post‐treatment, it was clear that cell clones with higher stemness exhibited an increased prevalence after treatment. This finding aligns with the previous understanding of the characteristics of chemotherapy resistance in leukaemia [46]. The significance of our study lies in the application of single‐cell multi‐omics analysis, which offers a unique opportunity to delve into the specific traits of these evolving T‐ALL cell clones, providing a more comprehensive understanding of the dynamics of drug‐resistant clonal evolution.

Drug treatment can induce changes in the transcription and chromatin accessibility landscape of tumour cells [47, 48]. We observed the expression and chromatin accessibility of PDCD1 and CXCR4 increased in T‐ALL cells post‐treatment, suggesting PDCD1 and CXCR4 inhibitors may be considered to be used for against refractory T‐ALL. A recent study has implicated PDCD1 as a marker gene for stemness of T‐ALL cells, and PD1^+^ T‐ALL cells showed high activity of MYC signalling and low activity of TCR signalling pathway [49]. Our findings also revealed elevated MYC pathway and decreased TCR pathway, along with stronger stemness in T‐ALL post‐treatment. Understanding these changes could provide valuable insights for developing targeted therapies in refractory T‐ALL.

In sum, our study revealed the presence of multiple malignant TCR clonotypes in T‐ALL patients at the single‐cell level, and ironically, some of these malignant TCR clonotypes likely corresponded to TCR gene rearrangement events in developing thymocytes rather than normal T cells, potentially pinpointing the developmental stage at which malignancy occurred. The T‐ALL cells with distinct TCR clonotypes showed subtle differences, particularly in cell stemness, which consequently influenced the overall prognosis of the disease. Remarkably, the T‐ALL cells with an orphan TCR β chain exhibited the highest stemness and displayed the highest resistance to drugs. These findings shed light on the heterogeneity of T‐ALL and emphasise the importance of considering the diversity of TCR clonotypes when assessing disease progression and treatment responses. Our study also improves the understanding of the mechanism underlying the generation of multiple TCR clonotypes and the potential role of TCR clonotype composition in driving the stem‐like properties of T‐ALL cells and their ability to evade conventional treatment methods.

## 4 Materials and Methods

### 4.1 Patient Recruitment and Sample Collection

This study was approved by the Ethical Committee of Jinan University (No. KY‐2019‐041). PB and BM of T‐ALL patients pre‐ and post‐treatment were collected for single‐cell sequencing. Clinical information of patients is shown in Table S6. PBMCs and BMMCs were isolated from samples with Ficoll.

### 4.2 Single‐Cell Library Preparation

scRNA‐seq and scTCR‐Seq libraries were constructed using the 10× Genomics Chromium Single Cell 5', and V(D)J enrichment reagent kit according to the manufacturer's protocols. Nuclear dissociation was performed to obtain scATAC‐seq libraries using the 10× Genomics Chromium Single Cell ATAC chemistry. Sequencing was performed with Illumina (NovaSeq 6000).

### 4.3 Pre‐Processing and Quality Filtering of scRNA‐Seq Datasets

The scRNA‐Seq raw reads were aligned to the GRCh38 reference genome using CellRanger v6.1.2. Subsequently, quality control and filtering were carried out by using Seurat v4.3.0. Genes expressed in less than 3 cells were excluded. Cells were filtered based on predefined criteria to ensure high‐quality data for downstream analysis. Specifically, cells with a minimum of 500 and a maximum of 5000 detected genes per cell were retained, along with the percentage of mitochondrial genes below 10%. Additionally, the removal of mitochondrial, and ribosomal genes was implemented.

### 4.4 Secondary Analysis of scRNA‐Seq Datasets

Seurat [50] v4.3.0 package was utilised for clustering and visualising the filtered scRNA‐Seq dataset. Gene expression values were normalised using the *NormalizeData* function, and the *FindVariableFeatures* function was used to select the top 2000 variable genes. In preparation for principal component analysis (PCA), the expression of genes in the dataset was subjected to scaling by the *ScaleData* function. Harmony [51] v0.1.1 package was employed for merging samples and removing batch effects. Clustering was performed by the *FindClusters* function. Dimensional reduction was carried out using UMAP, and subsequent annotation of each cluster was based on expression patterns of marker genes.

### 4.5 scTCR‐Seq Data Processing and Analysis

CellRanger v6.1.2 was employed to process the raw reads from scTCR‐Seq, utilising the ‘vdj’ pipeline and the V(D)J library of GRCh38. Cells with identical, UMI (unique molecular identifier) V, D, and J genes, and CDR3 sequences were defined as belonging to the same TCR clonotype. Cells were selectively retained based on specific clonotype criteria, including paired αβ chains or clonal expansion. scTCR‐Seq datasets were integrated with scRNA‐Seq datasets through cell barcodes.

### 4.6 scATAC‐Seq Data Processing and Analysis

CellRanger‐atac v2.0.0 was utilised to align the raw reads from scATAC‐Seq datasets to the GRCh38 reference genome. ArchR [52] v1.0.2 was used for quality control (QC) and downstream analyses. Following QC filtering, only cells meeting specific criteria were retained, including a transcription start site (TSS) enrichment score greater than 6 and a minimum of 3000 unique nuclear fragments. Dimensionality reduction was carried out using Iterative Latent Semantic Indexing, and clustering was implemented by utilising the *FindClusters* function within Seurat through the *addCluster* function within ArchR. Batch effects were removed by the *addHarmony* function. Cell clusters were annotated by marker gene scores, marker gene chromatin accessibility, and integration with our scRNA‐Seq datasets. Peak calling was performed by MACS2. TF binding motifs were introduced from the JASPAR 2020 database for analysis. Differential enrichment of motifs was identified through the utilisation of chromVAR. Pseudotime trajectory of normal T cells and tumour cells was analysed by the *addTrajectory* function. The co‐accessibility of genomic regions was defined by the *addPeak2GeneLinks* function. GREAT [53] was utilised for mapping and analysing gene‐enhancer relationships.

### 4.7 Random‐Forest Model for Distinguishing Malignant and Non‐Malignant Cells

The random‐forest model was employed to differentiate tumour and non‐tumour cells based on expression levels of TFs using the R package random‐Forest v4.7–1.1. The scRNA‐Seq dataset was randomly divided into a training set (70%) and a test set (30%). The *tuneRF* function was utilised to identify the optimal parameters, mtry (80) and ntrees (170).

### 4.8 Gene Regulatory Network Analysis

Pyscenic [54] v0.12.1 was used to perform gene regulatory network analysis. Only the regulon targets with weight ≥ 0.01 were retained. The inference of regulatory interactions between TFs and target genes was conducted employing the cisTarget module. Activities of regulons were identified by the AUCell algorithm. The DyNet algorithm was used to compare the regulon networks of normal T cells and tumour cells, aiming to investigate transcriptional rewiring of these networks due to malignancy.

### 4.9 JNU Dataset

PB samples from 27 de novo diagnosed T‐ALL patients in our clinical centre (JNU) between July 2009 and October 2023, were used as JNU dataset to assess TF expression levels. Additionally, PB samples from nine healthy donors were collected as a control group. The clinical information of T‐ALL patients, including median OS time, event, age, and gender, are listed in Table S7.

### 4.10 qRT‐PCR

PBMCs obtained from T‐ALL patients and healthy donors CD3+ T cells enriched using human CD3 microbeads (Miltenyi Biotec, Bergisch Gladbach, Germany) were processed with TRIzol reagent (Invitrogen, Carlsbad, California, USA) to extract RNA. RNA was reverse transcribed into cDNA by cDNA Reverse Transcription Kit (Applied Biosystems, Foster, CA, USA). The expression levels of mRNA for *KLF3*, *TCF3*, *TCF12*, *IKZF2*, and *ETS2* were detected using a qRT‐PCR kit (TIANGEN, Beijing, China) according to the manufacturer's instructions. The relative mRNA expression levels were normalised to β‐actin by the 2^−ΔΔCT^ method. The primers used for qRT‐PCR are shown in Table S8.

### 4.11 Survival Analysis

The optimal prognostic cut‐off values were calculated using X‐tile (version3.6.1, Yale University, New Haven, CT, USA) [55, 56]. Kaplan–Meier curves were conducted by using a survival package. Differences between subgroups were analysed using the log‐rank test. A *p* value less than 0.05 was considered to be statistically significant.

### 4.12 Single‐Cell Cell Cycle Stage Analysis

Cell cycle analysis was performed using the *CellCycleScoring* function based on predefined gene sets of cell cycle‐related genes in the Seurat package.

### 4.13 Single‐Cell Early Progenitor Phenotype Scoring

Early progenitor phenotype score was calculating by *AddModuleScore* function of Seurat based on early progenitor phenotype‐related gene set. The gene set contains marker genes of HSC (haematopoietic stem cell), MPP (multipotent progenitor), CLP (common lymphoid progenitor) from BLUEPRINT [57], and marker genes of thymocytes at DN, ISP and DP stage identified by *FindAllMarkers* based on thymocytes scRNA‐Seq dataset.

### 4.14 Tumour‐Associated Signalling Pathway Analysis

Tumour‐associated signalling pathway activity of tumour cells was defined by PROGENy [58] v1.16.0 based on the scRNA‐Seq dataset. Pathway activity scores were calculated and added to the Seurat object by *progeny*. ScaleData was used to standardise the signalling pathway scores. Then, we matched signalling pathway scores with different TCR clonotypes.

### 4.15 Gene Set Functional Enrichment Analysis

Functional enrichment analysis of marker genes was performed by cluster profile [59] v4.2.2 according to hallmark gene sets (H), curated gene sets (C2), and ontology gene sets (C5) from Molecular Signatures Database (MsigDB).

### 4.16 Pseudotime Trajectory Analysis of Tumour Cells

Tumour cells were clustered according to marker genes of HSC, MPP, CLP, and thymocytes at DN, ISP, and DP stages, respectively. The results of pseudotime trajectory were performed by scvelo [60] v0.2.5 and projected onto UMAP. To assess the developmental potential, CytoTRACE v.0.3.3 was utilised.

## Author Contributions

O.J.L., Y.L., C.Z. and R.F. contributed to the concept development, study design, and editing of the manuscript. S.S., X.W. and Y.Z. contributed equally to this work. S.S. performed experiments, analysed the data and wrote the manuscript. R.F., X.W. and H.Z. provided the clinical samples and treated the patients. Y.Z. helped with the experiments. Q.F., L.M. and B.H. assisted with the data interpretation. G.C. assisted with editing of the manuscript. X.Z., W.L. and H.C. assisted with the sample collection. All authors read and approved the final manuscript.

## Conflicts of Interest

The authors declare no conflicts of interest.

## Supporting information


**Figure S1.** Characterisation of T‐ALL cells in PBMCs and BMMCs of T‐ALL patients based on scRNA‐Seq and scTCR‐Seq. (A) Projection of expression levels of selected marker genes on UMAP visualisation. The UMAP consists of all PBMC cells from two T‐ALL patients and two healthy donors. (B) Contingency tables of the random‐forest model for predicting malignant T cells based on single‐cell transcriptome profiles with total (left), training (mid) and test (right) datasets. (C) The relative importance of TFs from the random‐forest model for predicting malignant T cells. (D) Boxplot comparison of expression levels of the top 5 ranked TFs from the random‐forest model between T‐ALL patients and healthy individuals. **p < 0.01; ***p < 0.001 (Wilcoxon rank‐sum test, two‐sided). (E) UMAP plot of PBMC scRNA‐seq datasets from two T‐ALL patients post‐treatment and two healthy donors, colour‐coded by five distinct cell types. (F) Dot plot of marker genes for each cell type in PBMCs of two T‐ALL patients post‐treatment and two healthy donors. (G) Same as (E), but colour‐coded by sample origin. P1: Patient1 post‐treatment; P2: Patient2 post‐treatment; HD1: Healthy donor 1; HD2: Healthy donor 2. (H) UMAP plots of BMMCs from two T‐ALL patients pre‐treatment, colour‐coded by individual TCR clonotype. Patient origin, clonotype ID, clonotype frequency and actual TCRαβ clonotype are shown on top of each UMAP. (I) Same as (H), but for post‐treatment of the two T‐ALL patients.

**Figure S2.** Additional characterisation of T‐ALL cells in PBMC and BMMC from T‐ALL patients based on scATAC‐Seq. (A) Heatmap visualisation of the fraction of cells in each PBMC cell type defined by scATAC‐Seq data that are annotated as the same cell type according to the corresponding scRNA‐Seq data. (B) Projection of gene scores of additional marker genes on PBMC scATAC‐Seq UMAP visualisation. Gene score reflects the predicted expression of the corresponding gene, a higher score implies higher accessibility. (C) Track view of the PBMC scATAC‐Seq data for additional loci in distinct cell types. (D) Heatmap visualisation of the fraction of cells in each BMMC cell type defined by scATAC‐Seq data that are annotated as the same cell type according to the corresponding scRNA‐Seq data. (E) Projection of gene scores of additional marker genes on BMMC scATAC‐Seq UMAP visualisation. (F) Track view of the BMMC scATAC‐Seq data for additional loci in distinct cell types.

**Figure S3.** Transcriptional reprogramming in T‐ALL cells. (A) Circos plots showing additional exemplary regulons in normal T cell and T‐ALL cell, respectively. Normal T‐cell‐specific and T‐ALL cell‐specific transcription factor (TF) is shown in blue and red, respectively, with links connecting to the target genes. The top 30 target genes are shown for each TF. The node size of target genes is proportional to regulation weight by the corresponding TF. (B) Regulons containing TFs and top targets activated in both normal T cells and T‐ALL cells, such as *ETS2* (left) and *IKZF2* (right). The top 10 target genes for *ETS2* (left) and *IKZF2* (right) in normal T cells and T‐ALL cells are depicted with blue and red links, respectively. Yellow links indicate the top 10 target genes uniquely found in T‐ALL cells compared to normal T cells. (C) Boxplots for comparison of *KLF6*, and *TCF3* expression levels between normal T cells and T‐ALL cells. (D) Boxplots for comparison of *ETS2*, and *IKZF2* expression levels between normal T cells and T‐ALL cells. (E) Kaplan–Meier curves for the co‐expression status of KLF3 and TCF3 in predicting OS of T‐ALL patients from the JNU dataset. (F) Kaplan–Meier curves for the co‐expression status of KLF3 and TCF3 in predicting EFS of T‐ALL patients from the TARGET database.

**Figure S4.** Differential chromatin accessibility landscape between T‐ALL cells and normal T cells based scATAC‐Seq data. (A) Bar chart showing the number of chromatin accessibility peaks identified in T‐ALL cells, normal T cells, and their union. (B) Summary of a number of differential chromatin accessibility peaks in normal T cells or T‐ALL cells (FDR ≤ 0.1, absolute log2FC ≥ 1). (C) Heatmap showing the scATAC‐Seq signals around transcription start sites (TSSs) with differential chromatin accessibility peaks in normal T cells and T‐ALL cells. (D) Boxplots for comparing the distance between chromatin accessibility peaks to the nearest TSS between normal T cells and T‐ALL cells. ***p < 0.001 (Wilcoxon rank‐sum test, two‐sided). (E) Boxplots for comparing the number of genes related to ≥ 15 enhancers between normal T cells and T‐ALL cells. ***p < 0.001 (Wilcoxon rank‐sum test, two‐sided). (F) Track view of scATAC‐Seq data, chromatin accessibility peaks and predicted peak‐to‐gene interaction of additional exemplary loci.

**Figure S5.** Comparison of T‐ALL cells in peripheral blood and bone marrow. (A) Violin plots illustrating the expression levels of *SPINK2* (left) or *AC002454.1* (right) in thymocytes originating from different differentiation stages. (B) Violin plots showing the expression levels of *PTCRA* (left) or *MZB1* (right) in thymocytes originating from different differentiation stages. (C) UMAP plots of T‐ALL cells from the scRNA‐seq dataset colour‐coded by the individual of peripheral blood and bone marrow in Patient1 (top) or Patient2 (bottom). (D) Volcano plots for marker genes of T‐ALL cells from peripheral blood and bone marrow in Patient1 (top) or Patient2 (down) based on scRNA‐seq. (E) Volcano plots for marker genes of T‐ALL cells divided by different TCR clonotypes from peripheral blood and bone marrow in Patient1 (top) or Patient2 (bottom) based on scRNA‐seq. (F) UMAP plots of T‐ALL cells from the scATAC‐seq dataset colour‐coded by an individual of peripheral blood and bone marrow in Patient1 (top) or Patient2 (bottom). (G) Stacked barplot showing the fraction of different types of peaks in T‐ALL cells from peripheral blood and bone marrow in Patient1 (top) or Patient2 (bottom). (H) Volcano plots for marker peaks of T‐ALL cells from peripheral blood and bone marrow in Patient1 (top) or Patient2 (bottom) based on the scATAC‐Seq dataset.

**Figure S6.** Differences between root cells and endpoint cells identified by RNA velocity. (A) Gene set enrichment analysis (GSEA) plot showing the expression enrichment of genes related to haematopoietic stem cell pathway in root cells. (B) GSEA plot showing the expression enrichment of genes related to T‐lymphocyte differentiation (left) and interferon alpha response (right) pathway in the endpoint cells.an (C) Proportion of root and endpoint cells in different cell cycle phases. (D) Heatmap displaying the expression levels of marker genes of root and endpoint cells.

**Figure S7.** Additional pre‐ and post‐treatment T‐ALL cell comparison. (A) Volcano plots showing the differentially expressed genes (left) and surface protein genes (right) between Patient2 pre‐ and post‐treatment T‐ALL cells divided by different TCR clonotypes. (B) Browser tracks showing the chromatin accessibility profile and peak‐to‐gene links at the *RGCC* locus (left) and *NR4A3* locus (right) across T‐ALL cells pre‐ and post‐treatment of Patient2.

**Table S1.** scRNA‐Seq dataset information after QC.

**Table S2.** scTCR‐Seq dataset information.

**Table S3.** scATAC‐Seq dataset information after QC.

**Table S4.** Dynet rewiring score.

**Table S5.** Enriched pathways for the upregulated genes in the five different TCR clonotypes.

**Table S6.** Clinical features of the two patients for single‐cell sequencing.

**Table S7.** Clinical characteristics of T‐ALL patients.

**Table S8.** Sequences of primers used in qRT—PCR.

## Data Availability

The raw sequence data of patients reported in this paper have been deposited in the Genome Sequence Archive in National Genomics Data Center, China National Center for Bioinformation/Beijing Institute of Genomics, Chinese Academy of Sciences (GSA‐Human: HRA005267) that are publicly accessible at https://ngdc.cncb.ac.cn/gsa‐human. Datasets of PMBCs from healthy donors were obtained from GSE157007 and GSE175694. Datasets of thymocytes were downloaded from GSE195812. Microarray data of T‐ALL patients and healthy donors was acquired from GSE13159.
