# Supplementary material for: Single‐Cell Multiomics Reveals TCR Clonotype‐Specific Phenotype and Stemness Heterogeneity of T‐ALL Cells
Source: Cell Prolif. 2024 Dec 15;58(4):e13786. doi: 10.1111/cpr.13786 (PMC11969251; doi:10.1111/cpr.13786)

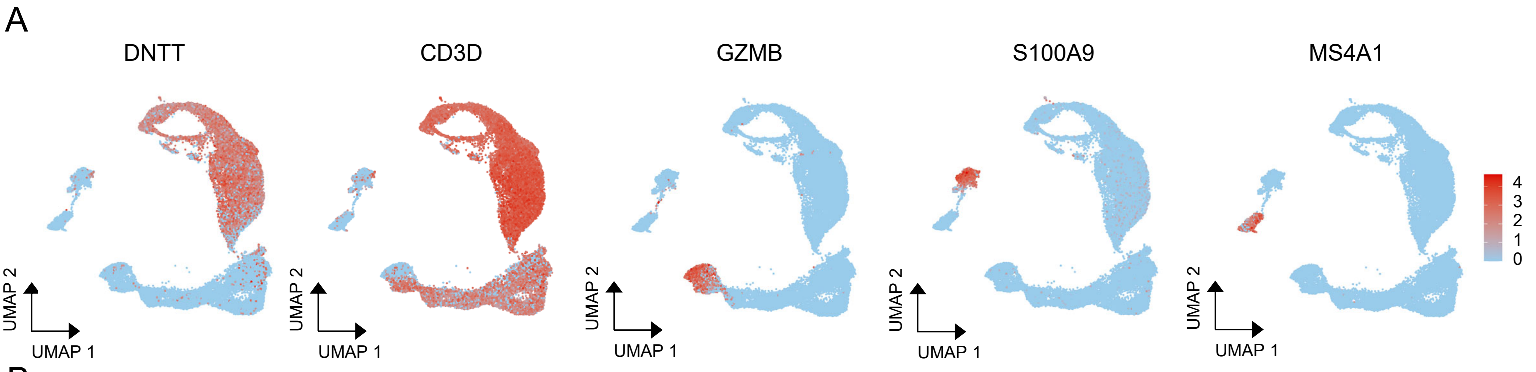

B

	Malignant	Non-malignant	Class error
Malignant	10595	133	0.012
Non-malignant	207	9446	0.021

Predict_ train	Malignant	Non-malignant
Malignant	10728	0
Non-malignant	0	9653

Predict_ test	Malignant	Non-malignant
Malignant	4502	56
Non-malignant	72	4106

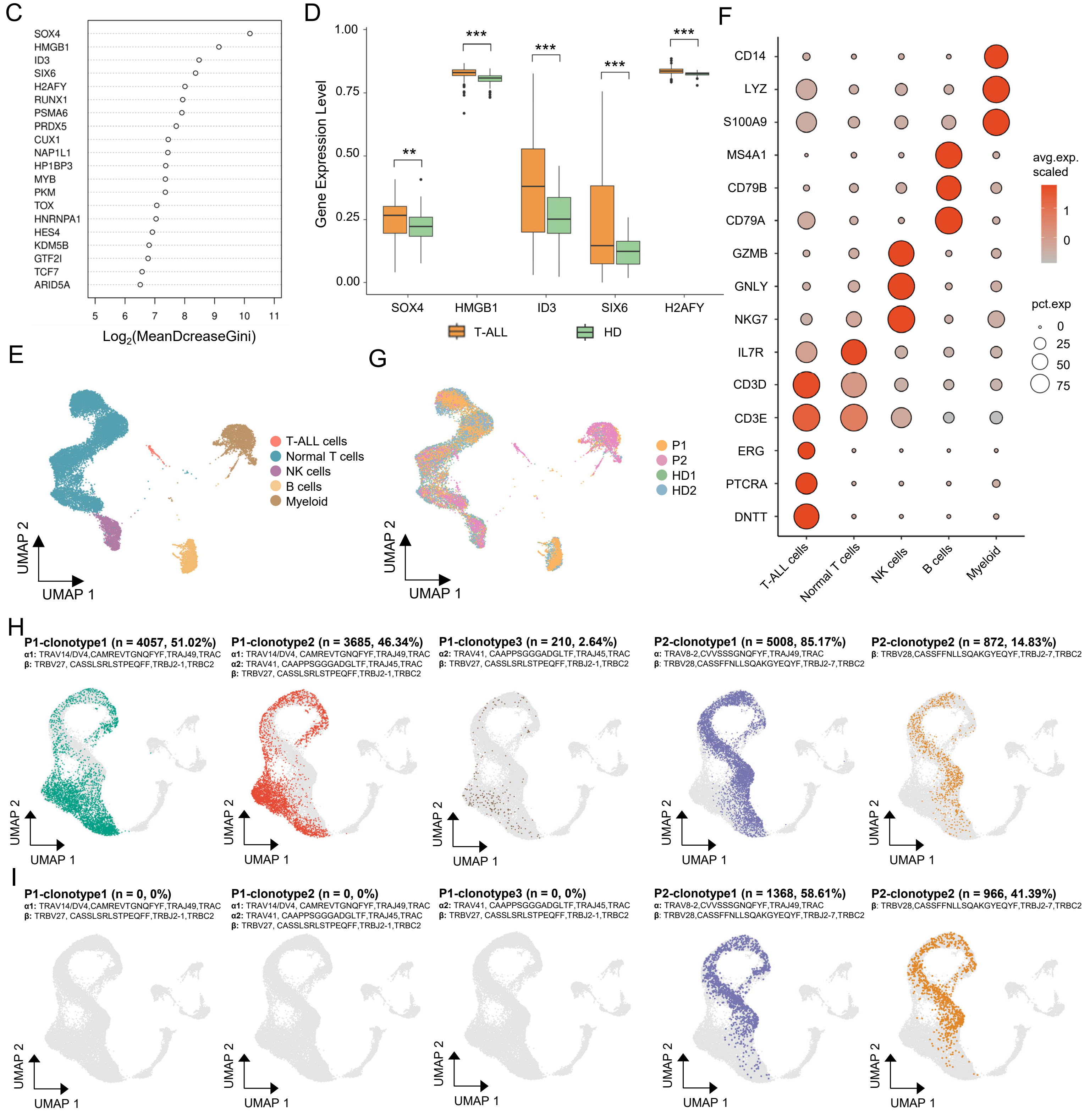

Supplement: Supplementary file 1 — Figure S1. Characterisation of T‐ALL cells in PBMCs and BMMCs of T‐ALL patients based on scRNA‐Seq and scTCR‐Seq. (A) Projection of expression levels of selected marker genes on UMAP visualisation. The UMAP consists of all PBMC cells from two T‐ALL patients and two healthy donors. (B) Contingency tables of the random‐forest model for predicting malignant T cells based on single‐cell transcriptome profiles with total (left), training (mid) and test (right) datasets. (C) The relative importance of TFs from the random‐forest model for predicting malignant T cells. (D) Boxplot comparison of expression levels of the top 5 ranked TFs from the random‐forest model between T‐ALL patients and healthy individuals. **p < 0.01; ***p < 0.001 (Wilcoxon rank‐sum test, two‐sided). (E) UMAP plot of PBMC scRNA‐seq datasets from two T‐ALL patients post‐treatment and two healthy donors, colour‐coded by five distinct cell types. (F) Dot plot of marker genes for each cell type in PBMCs of two T‐ALL patients post‐treatment and two healthy donors. (G) Same as (E), but colour‐coded by sample origin. P1: Patient1 post‐treatment; P2: Patient2 post‐treatment; HD1: Healthy donor 1; HD2: Healthy donor 2. (H) UMAP plots of BMMCs from two T‐ALL patients pre‐treatment, colour‐coded by individual TCR clonotype. Patient origin, clonotype ID, clonotype frequency and actual TCRαβ clonotype are shown on top of each UMAP. (I) Same as (H), but for post‐treatment of the two T‐ALL patients. [file CPR-58-e13786-s006.pdf]
