# Supplementary figures and images for: Single‐Cell Multiomics Reveals TCR Clonotype‐Specific Phenotype and Stemness Heterogeneity of T‐ALL Cells
Source: Cell Prolif. 2024 Dec 15;58(4):e13786. doi: 10.1111/cpr.13786 (PMC11969251; doi:10.1111/cpr.13786)

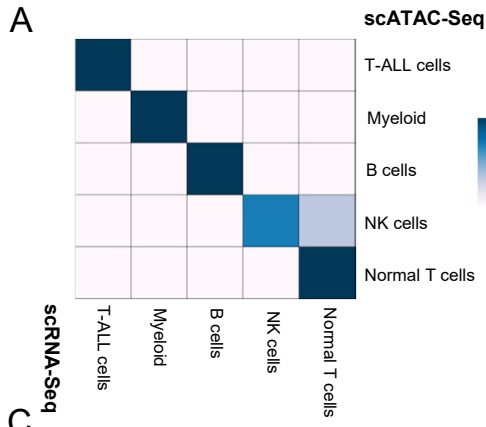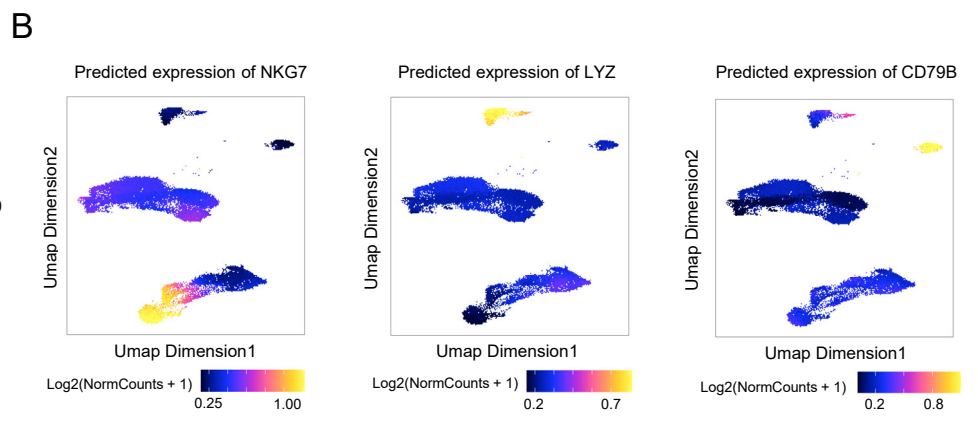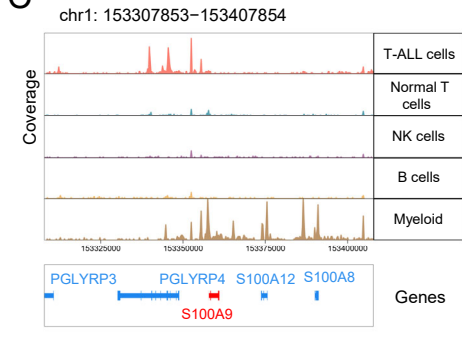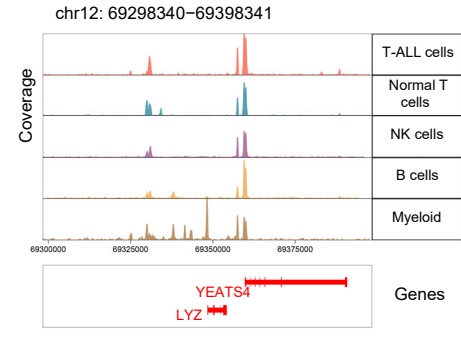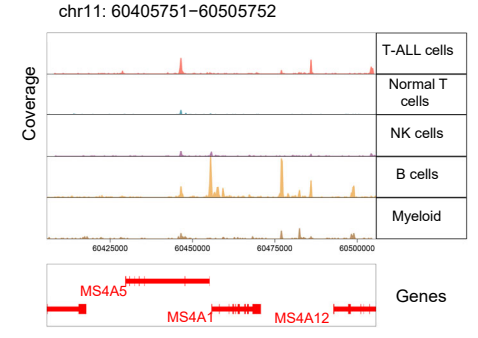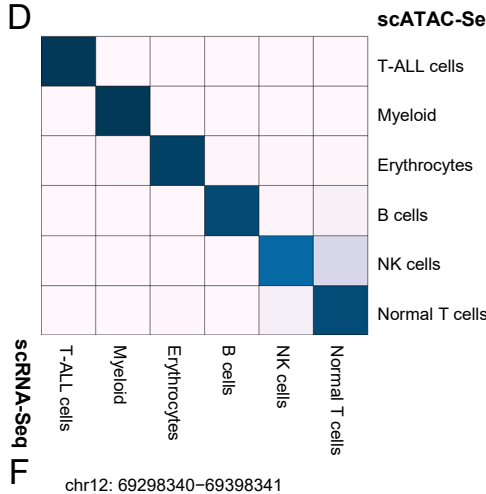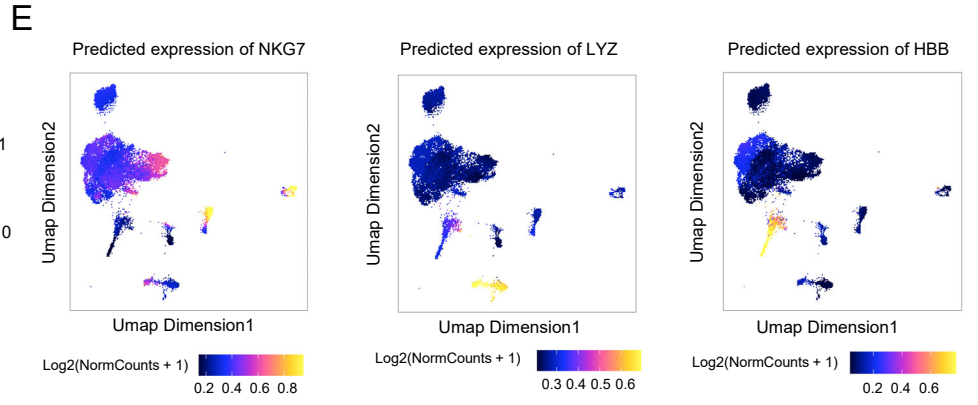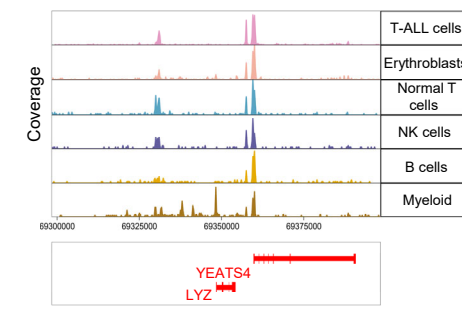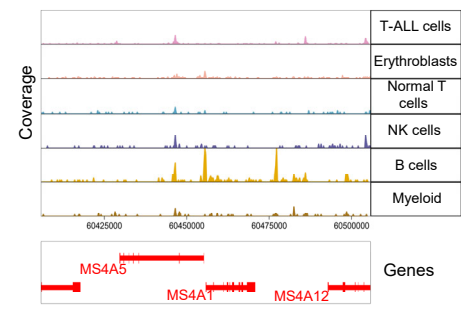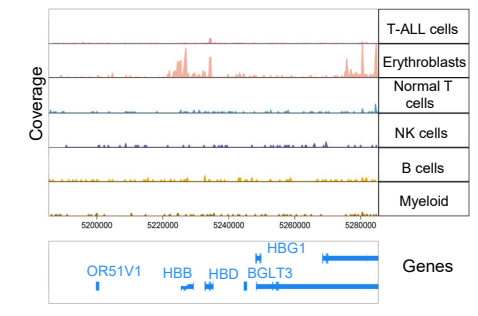

Supplement: Supplementary file 2 — Figure S2. Additional characterisation of T‐ALL cells in PBMC and BMMC from T‐ALL patients based on scATAC‐Seq. (A) Heatmap visualisation of the fraction of cells in each PBMC cell type defined by scATAC‐Seq data that are annotated as the same cell type according to the corresponding scRNA‐Seq data. (B) Projection of gene scores of additional marker genes on PBMC scATAC‐Seq UMAP visualisation. Gene score reflects the predicted expression of the corresponding gene, a higher score implies higher accessibility. (C) Track view of the PBMC scATAC‐Seq data for additional loci in distinct cell types. (D) Heatmap visualisation of the fraction of cells in each BMMC cell type defined by scATAC‐Seq data that are annotated as the same cell type according to the corresponding scRNA‐Seq data. (E) Projection of gene scores of additional marker genes on BMMC scATAC‐Seq UMAP visualisation. (F) Track view of the BMMC scATAC‐Seq data for additional loci in distinct cell types. [file CPR-58-e13786-s004.pdf]

A

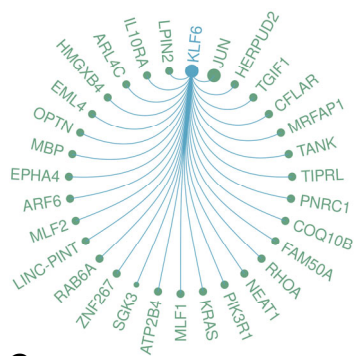

B

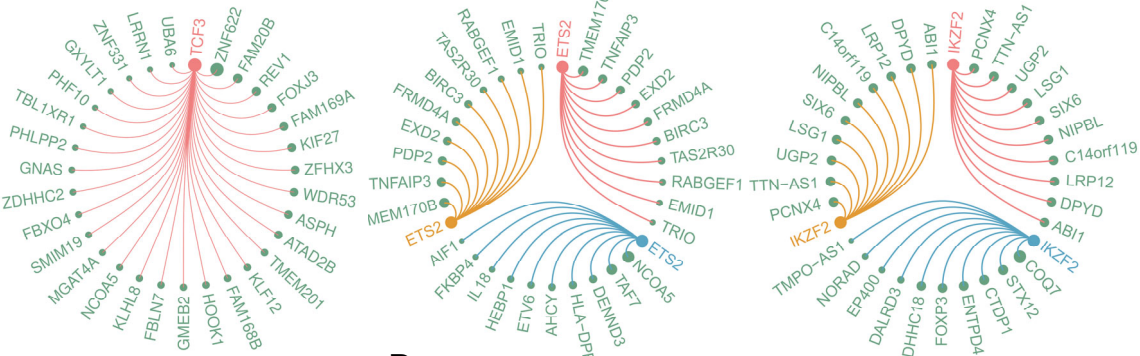

C

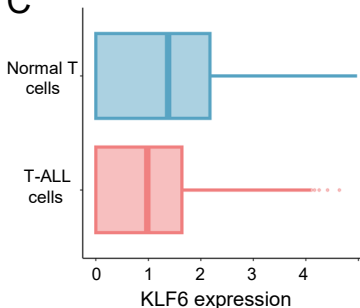

D

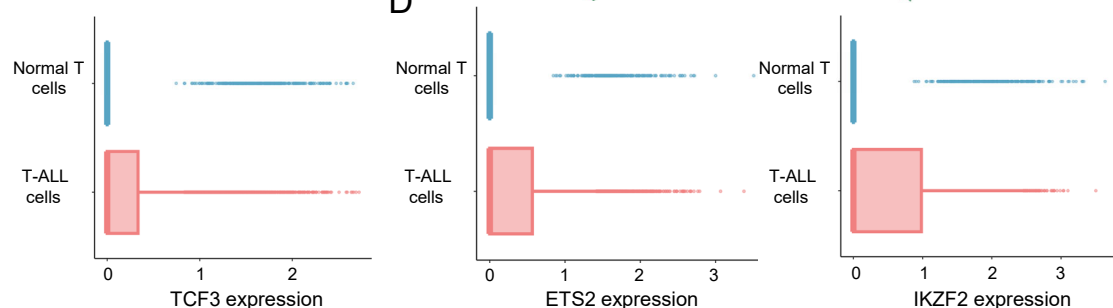

E

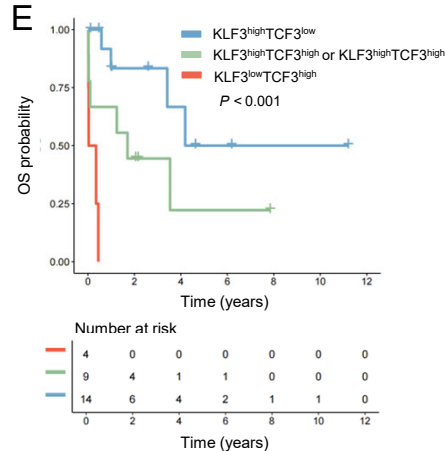

F

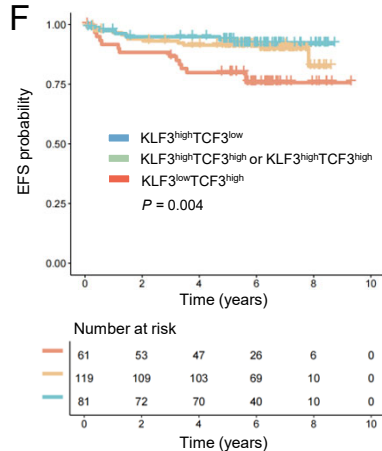

Supplement: Supplementary file 3 — Figure S3. Transcriptional reprogramming in T‐ALL cells. (A) Circos plots showing additional exemplary regulons in normal T cell and T‐ALL cell, respectively. Normal T‐cell‐specific and T‐ALL cell‐specific transcription factor (TF) is shown in blue and red, respectively, with links connecting to the target genes. The top 30 target genes are shown for each TF. The node size of target genes is proportional to regulation weight by the corresponding TF. (B) Regulons containing TFs and top targets activated in both normal T cells and T‐ALL cells, such as ETS2 (left) and IKZF2 (right). The top 10 target genes for ETS2 (left) and IKZF2 (right) in normal T cells and T‐ALL cells are depicted with blue and red links, respectively. Yellow links indicate the top 10 target genes uniquely found in T‐ALL cells compared to normal T cells. (C) Boxplots for comparison of KLF6, and TCF3 expression levels between normal T cells and T‐ALL cells. (D) Boxplots for comparison of ETS2, and IKZF2 expression levels between normal T cells and T‐ALL cells. (E) Kaplan–Meier curves for the co‐expression status of KLF3 and TCF3 in predicting OS of T‐ALL patients from the JNU dataset. (F) Kaplan–Meier curves for the co‐expression status of KLF3 and TCF3 in predicting EFS of T‐ALL patients from the TARGET database. [file CPR-58-e13786-s013.pdf]

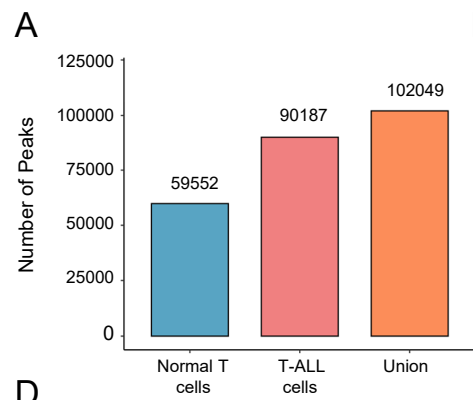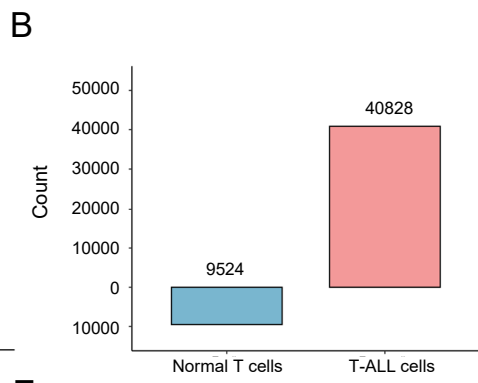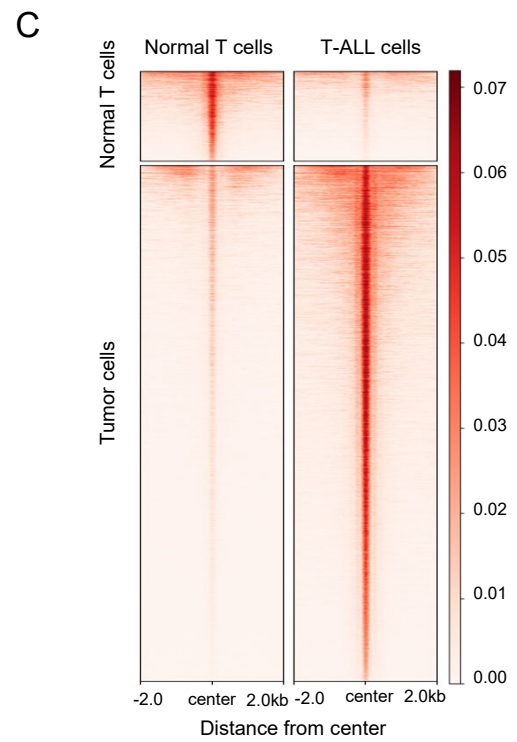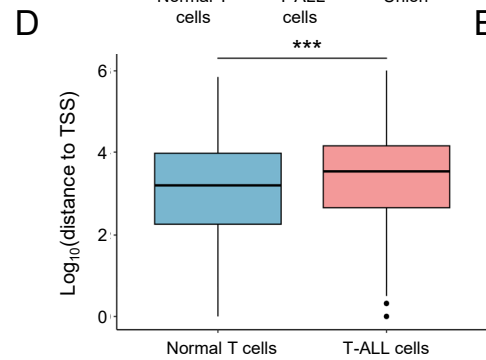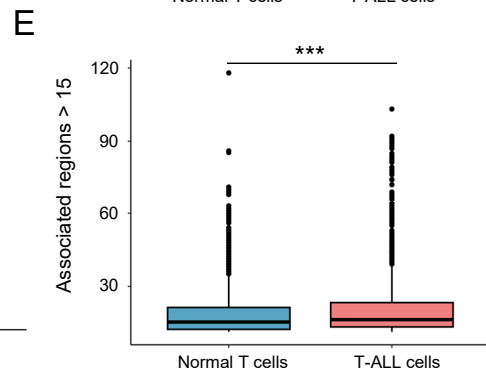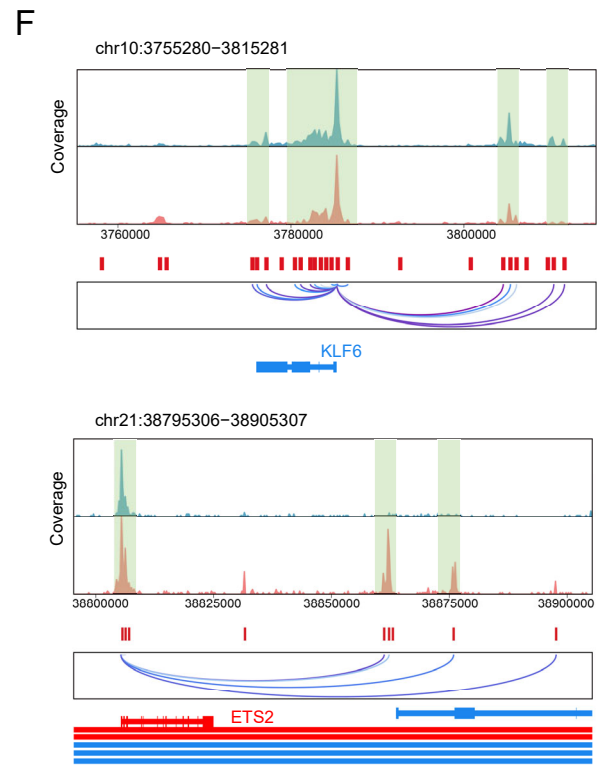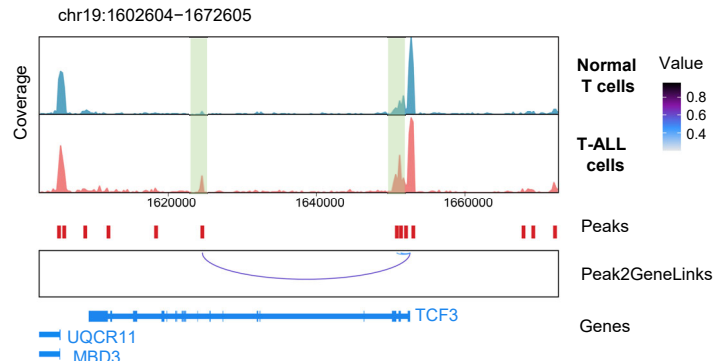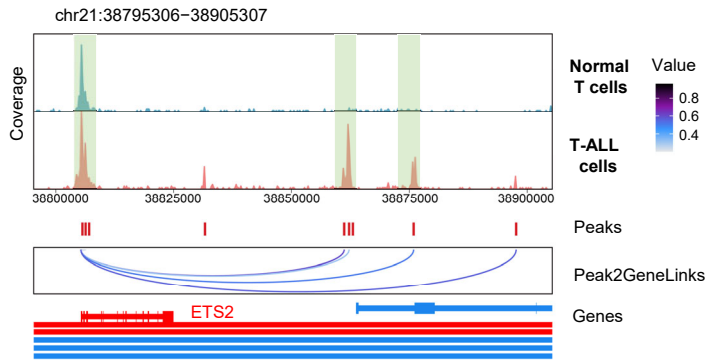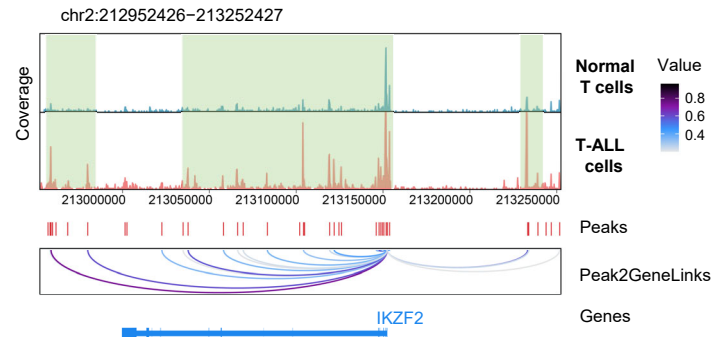

Supplement: Supplementary file 4 — Figure S4. Differential chromatin accessibility landscape between T‐ALL cells and normal T cells based scATAC‐Seq data. (A) Bar chart showing the number of chromatin accessibility peaks identified in T‐ALL cells, normal T cells, and their union. (B) Summary of a number of differential chromatin accessibility peaks in normal T cells or T‐ALL cells (FDR ≤ 0.1, absolute log2FC ≥ 1). (C) Heatmap showing the scATAC‐Seq signals around transcription start sites (TSSs) with differential chromatin accessibility peaks in normal T cells and T‐ALL cells. (D) Boxplots for comparing the distance between chromatin accessibility peaks to the nearest TSS between normal T cells and T‐ALL cells. ***p < 0.001 (Wilcoxon rank‐sum test, two‐sided). (E) Boxplots for comparing the number of genes related to ≥ 15 enhancers between normal T cells and T‐ALL cells. ***p < 0.001 (Wilcoxon rank‐sum test, two‐sided). (F) Track view of scATAC‐Seq data, chromatin accessibility peaks and predicted peak‐to‐gene interaction of additional exemplary loci. [file CPR-58-e13786-s011.pdf]

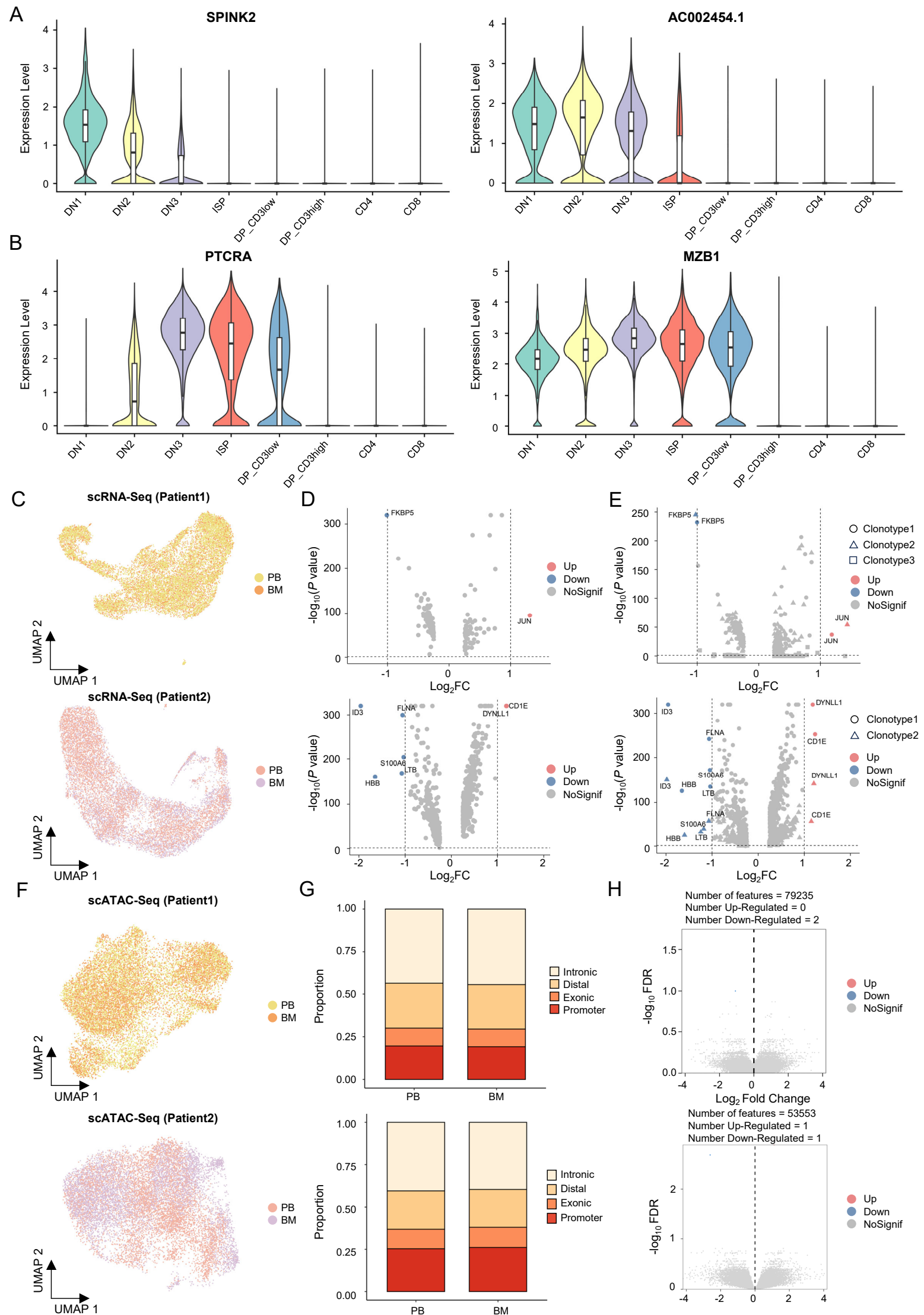

Supplement: Supplementary file 5 — Figure S5. Comparison of T‐ALL cells in peripheral blood and bone marrow. (A) Violin plots illustrating the expression levels of SPINK2 (left) or AC002454.1 (right) in thymocytes originating from different differentiation stages. (B) Violin plots showing the expression levels of PTCRA (left) or MZB1 (right) in thymocytes originating from different differentiation stages. (C) UMAP plots of T‐ALL cells from the scRNA‐seq dataset colour‐coded by the individual of peripheral blood and bone marrow in Patient1 (top) or Patient2 (bottom). (D) Volcano plots for marker genes of T‐ALL cells from peripheral blood and bone marrow in Patient1 (top) or Patient2 (down) based on scRNA‐seq. (E) Volcano plots for marker genes of T‐ALL cells divided by different TCR clonotypes from peripheral blood and bone marrow in Patient1 (top) or Patient2 (bottom) based on scRNA‐seq. (F) UMAP plots of T‐ALL cells from the scATAC‐seq dataset colour‐coded by an individual of peripheral blood and bone marrow in Patient1 (top) or Patient2 (bottom). (G) Stacked barplot showing the fraction of different types of peaks in T‐ALL cells from peripheral blood and bone marrow in Patient1 (top) or Patient2 (bottom). (H) Volcano plots for marker peaks of T‐ALL cells from peripheral blood and bone marrow in Patient1 (top) or Patient2 (bottom) based on the scATAC‐Seq dataset. [file CPR-58-e13786-s014.pdf]

A

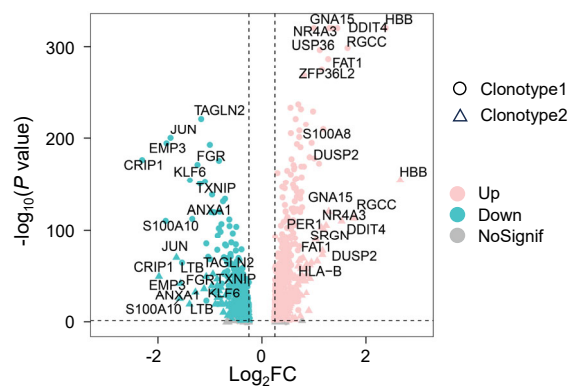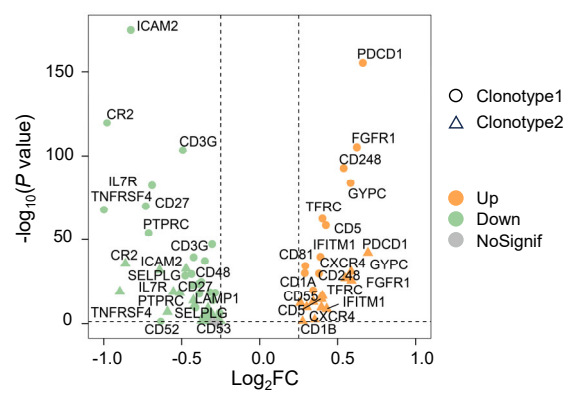

B

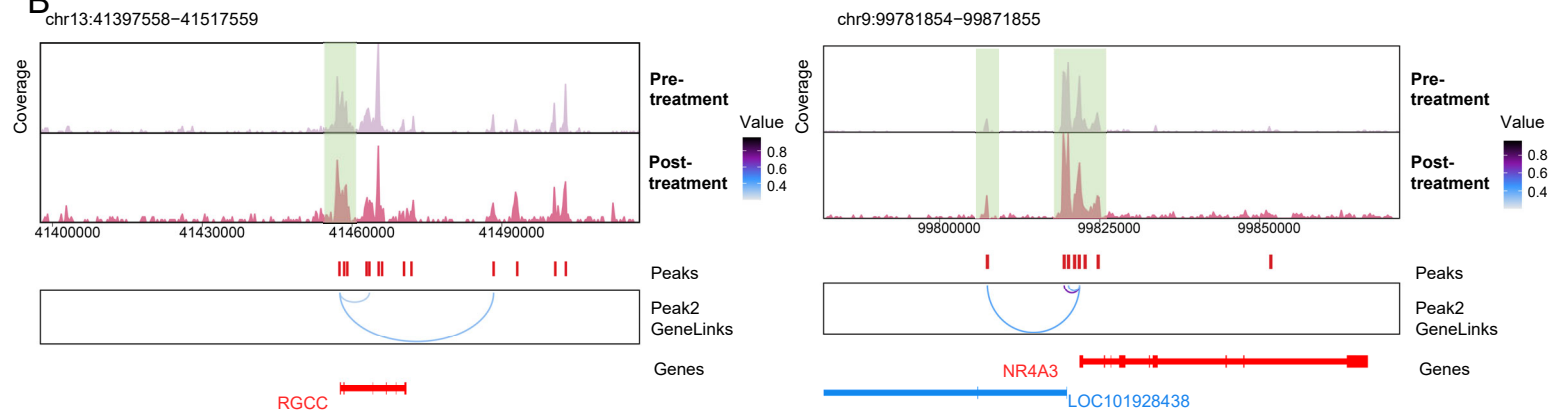

Supplement: Supplementary file 7 — Figure S7. Additional pre‐ and post‐treatment T‐ALL cell comparison. (A) Volcano plots showing the differentially expressed genes (left) and surface protein genes (right) between Patient2 pre‐ and post‐treatment T‐ALL cells divided by different TCR clonotypes. (B) Browser tracks showing the chromatin accessibility profile and peak‐to‐gene links at the RGCC locus (left) and NR4A3 locus (right) across T‐ALL cells pre‐ and post‐treatment of Patient2. [file CPR-58-e13786-s015.pdf]
