# Supplementary material for: Single‐Cell Multiomics Reveals TCR Clonotype‐Specific Phenotype and Stemness Heterogeneity of T‐ALL Cells
Source: Cell Prolif. 2024 Dec 15;58(4):e13786. doi: 10.1111/cpr.13786 (PMC11969251; doi:10.1111/cpr.13786)
Supplement: Supplementary file 6 — Figure S6. Differences between root cells and endpoint cells identified by RNA velocity. (A) Gene set enrichment analysis (GSEA) plot showing the expression enrichment of genes related to haematopoietic stem cell pathway in root cells. (B) GSEA plot showing the expression enrichment of genes related to T‐lymphocyte differentiation (left) and interferon alpha response (right) pathway in the endpoint cells.an (C) Proportion of root and endpoint cells in different cell cycle phases. (D) Heatmap displaying the expression levels of marker genes of root and endpoint cells. [file CPR-58-e13786-s002.pdf]

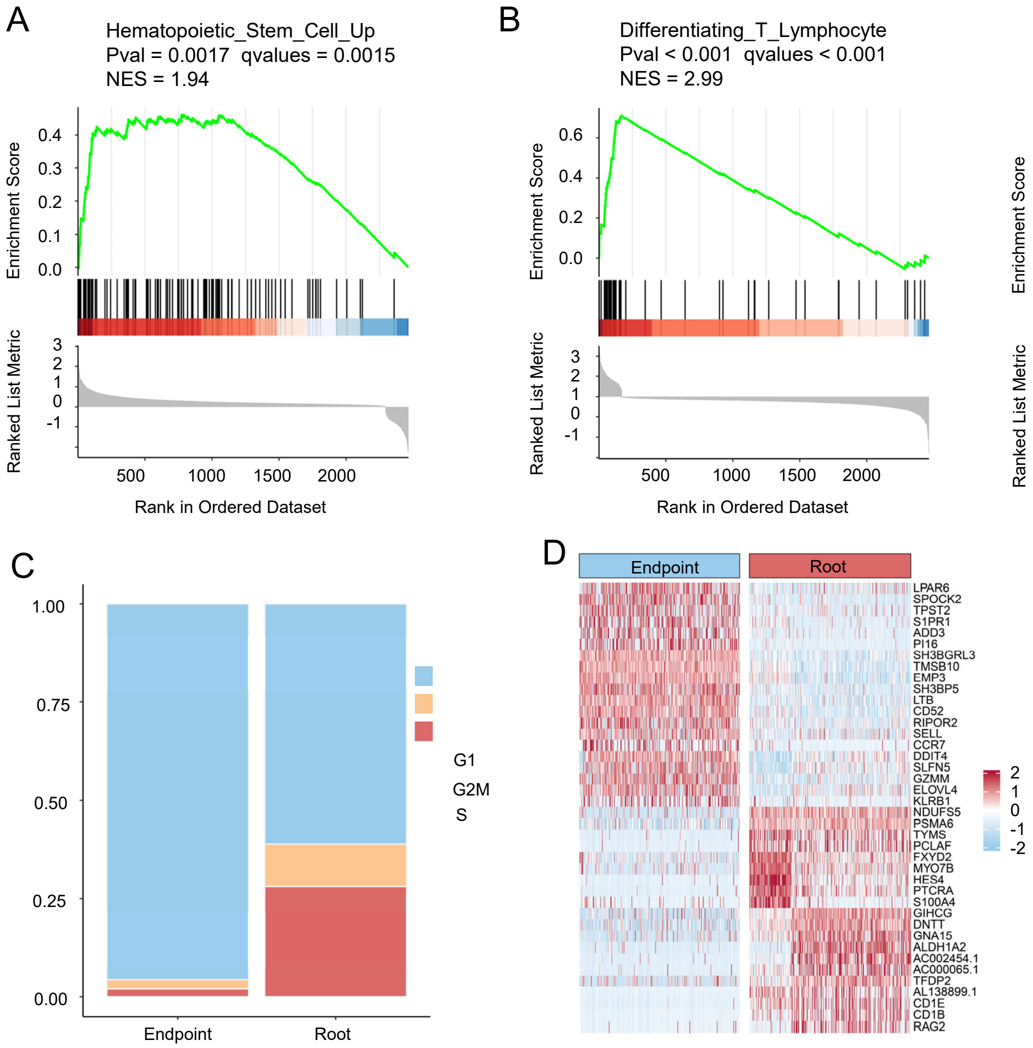
A
Hematopoietic_Stem_Cell_Up
Pval = 0.0017 qvalues = 0.0015
NES = 1.94
B
Differentiating_T_Lymphocyte
Pval < 0.001 qvalues < 0.001
NES = 2.99
Interferon_Alpha_Response
Pval < 0.001 qvalues < 0.001
NES = 2.60
Enrichment Score
Ranked List Metric
Rank in Ordered Dataset
C
Proportion
G1
G2M
S
Endpoint
Root
D
Endpoint
Root
LPAR6
SPOCK2
TPST2
S1PR1
ADD3
PI16
SH3BGRL3
TMSB10
EMP3
SH3BP5
LTB
CD52
RIPOR2
SELL
CCR7
DDIT4
SLFN5
GZMM
ELOVL4
KLRB1
NDUFS5
PSMA6
TYMS
PCLAF
FXYD2
MYO7B
HES4
PTCRA
S100A4
GIHCG
DNTT
GNA15
ALDH1A2
AC002454.1
AC000065.1
TFDP2
AL138899.1
CD1E
CD1B
RAG2
